# Neonatal infection with *Bordetella pertussis* promotes autism-like phenotypes in mice

**DOI:** 10.1016/j.isci.2024.111548

**Published:** 2024-12-09

**Authors:** Eoin O’Neill, Lucy Curham, Caitlín Ní Chasaide, Síofra O’Brien, Gavin McManus, Barry Moran, Keith Rubin, Steven Glazer, Marina A. Lynch, Kingston H.G. Mills

**Affiliations:** 1 Immune Regulation Research Group, School of Biochemistry and Immunology, Trinity Biomedical Sciences Institute, Trinity College Dublin, D02R590 Dublin, Ireland; 2 Trinity College Institute of Neuroscience, Trinity College Dublin, D02PD91 Dublin, Ireland; 3 ILiAD Biotechnologies, Weston, FL 33331, USA

**Keywords:** Natural sciences, Biological sciences, Neuroscience, Behavioral neuroscience

## Abstract

Autism spectrum disorder (ASD) has been linked with infections early in life. Here we demonstrate that the infection of neonatal mice with the respiratory pathogen *Bordetella pertussis* leads to neuroinflammation, neurodevelopmental defects, and ASD-like behaviors. Following the respiratory challenge of neonatal mice with *B. pertussis,* multiple atypical CNS findings were observed*,* including blood-brain barrier disruption, dissemination of live *B. pertussis* bacteria to the brain with the concomitant infiltration of inflammatory monocytes, neutrophils, and activated IL-17A- and IFN-γ-producing CD4 T cells. Microglia from infected mice were activated, with impaired phagocytic function, resulting in defective synaptic pruning and disrupted neuronal circuit formation. Impaired neurodevelopment in *B. pertussis*-infected post-natal mice was associated with ASD-like behavioral abnormalities in young adulthood. Our data indicate that infection with virulent *B. pertussis* during infancy increases the risk of autism-like behavior in young adult mice. A study into the potential role of *B. pertussis* in human ASD is warranted.

## Highlights

• Live *B. pertussis* bacteria disseminate to the brain of infected neonatal mice
• Inflammatory immune cells infiltrate the brain of neonatal infected mice
• Microglia in *B. pertussis* infected mice have defective synaptic pruning
• Infection with *B. pertussis* during infancy leads to ASD-like behavior in adulthood

## Graphical abstract

**Figure:**
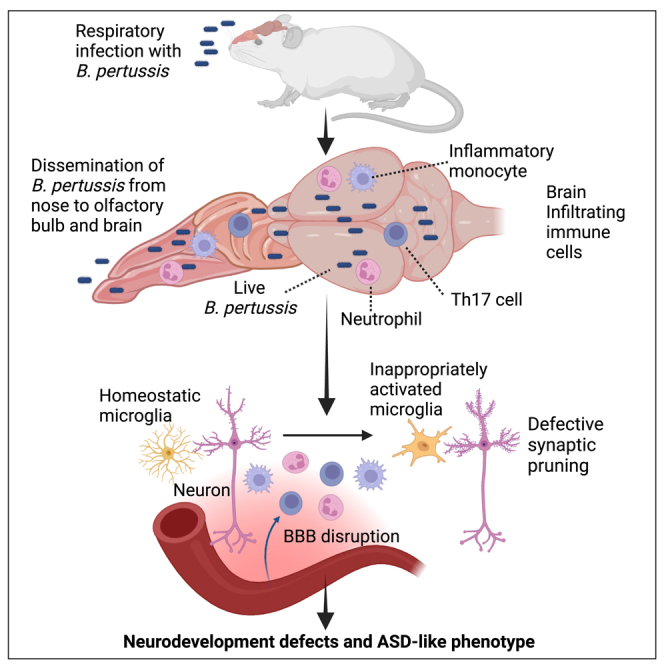

## Introduction

Autism spectrum disorder (ASD) is a complex neurodevelopmental disorder defined by social and communication deficits, stereotypic repetitive patterns of behavior, and restricted interests. The incidence, prevalence, and global burden of ASD has risen steadily over the past 30 years, suggesting a role for environmental factors in ASD etiology.^1^ Since ASD symptomatology typically manifests in early childhood, potential environmental causative factors, such as severe infection,^2^ would likely occur during the prenatal and early postnatal periods. Indeed, maternal viral infection in the first trimester and maternal bacterial infection during the second trimester of pregnancy are associated with an increased likelihood of ASD diagnosis in offspring,^3^ and children with ASD are more likely to have had neonatal or early childhood infections compared to the general population.^4^

The Gram-negative bacterium, *B. pertussis,* causes whooping cough (pertussis), a respiratory disease that is especially severe in young infants, for whom it can be lethal. Despite broad vaccine coverage, there has been a resurgence of pertussis in recent years, and it remains a public health threat worldwide.^5^ In diverse and highly *B. pertussis*-vaccinated countries, asymptomatic nasopharyngeal colonization with *B. pertussis* has been documented in 4.8–7.1% of individuals by PCR on nasal swabs^6^^,^^7^ and in 4.8.-14.1% by serology indicative of infection in the past year.^8^^,^^9^^,^^10^ Though current strategies for *B. pertussis* immunization reduce the risk of serious respiratory disease for a limited duration, they fail to prevent the infection of the nasal tract.^11^^,^^12^

Certain toxins secreted by *B. pertussis* have been shown to have neurobiological effects. For example, pertussis toxin (PT) disrupts the blood-brain barrier (BBB), inhibits neural growth cone guidance^13^ and neurite outgrowth,^14^ impairs neuroepithelial proliferation,^15^ and inhibits the release of brain-derived neurotrophic factor,^16^ which plays an important role during brain development.^17^ Furthermore, dermonecrotic toxin (DNT) is a neurotropic virulence factor produced by *B. pertussis* that affects neurons by binding voltage-gated Ca^2+^ channels, which are highly expressed in the central nervous system (CNS).^18^

Reports of neurological sequelae of critical pertussis during infancy include seizures, acute demyelination, pertussis-related encephalopathy,^19^^,^^20^ lower IQ scores and poorer school achievement,^21^ and long-term communication deficits.^22^ However, research addressing neurodevelopmental outcomes of children with pertussis is limited. Interestingly, the incidence of ASD in Sweden increased in children between 1984-1994,^23^ during a period with a high and increasing prevalence of pertussis following the cessation of the Swedish whole cell pertussis vaccination program in 1979.^24^ After the reintroduction of pertussis immunization in the 1990s, the incidence of pertussis and ASD prevalence rates declined. This abrupt fall in ASD rates contrasted markedly with worldwide ASD trends during the same period, confounding expected increases in ASD recognition, diagnosis, and reporting over time.^25^^,^^26^ Despite high acellular pertussis vaccination coverage, rates of pertussis and autism have concurrently risen in recent decades in many other developed nations, including the US.^27^^,^^28^

In a large prospective cohort study that assessed the cognitive development of infants one year after critical pertussis, 37% of infants with severe pertussis had neurodevelopmental deficits, with 9% having an early learning composite score of 2 or more standard deviations below population norms.^22^ Neurodevelopmental communication deficits included regression in age-appropriate competence in visual reception, vocalization, and facial expressiveness 1 year after severe *B. pertussis* illness. No other neurodevelopmental domains, such as motor function, feeding, or arousal regressed after infection, and illness severity did not correlate with neurodevelopmental scores and so does not support the notion that the severity of illness explains the selective deficits. Despite these associations and the epidemiological observations in Sweden, no studies to date have investigated the potential causal and mechanistic relationship between early life respiratory infection with *B. pertussis* and autism.

Based on the hypothesis that ASD may be the late clinical manifestation of an infant or early childhood clinical or subclinical *Bordetella pertussis* infection (Rubin and Glazer, article in preparation) and the evidence discussed above, we investigated whether neonatal respiratory infection with *B. pertussis* impacts neurodevelopment. Here we show that the respiratory challenge of neonatal mice with *B. pertussis* leads to multiple atypical CNS findings. We document live *B. pertussis* in the brain with the concomitant CNS infiltration of neutrophils, inflammatory monocytes, activated CD4 T cells, local activation of microglia with reduced phagocytic activity, and impaired early postnatal synaptic pruning of developing neuronal circuits. Our results indicate that in neonatal mice, respiratory infection with *B. pertussis* can lead to significant neurodevelopmental changes and impairment, with ASD-related behaviors later in life. Our study elucidates a mechanism by which infant infection with *B. pertussis* may increase the risk of autism.

## Results

### Bacteria in the brain and neuroinflammation following the respiratory infection of neonatal mice with *B. pertussis*

*B. pertussis* infects the lungs and nasal cavity of humans and mice, and the bacteria are thought not to disseminate from the respiratory tract. However, live bacteria have been detected in the liver following *B. pertussis* respiratory challenge of immunodeficient adult mice^29^ and in the spleen, liver, and blood of neonatal mice.^30^^,^^31^ Here we explored the possibility that in neonatal mice *B. pertussis* may disseminate to the brain or that immune cells activated by infection in the respiratory tract may infiltrate the brain and that this may impact neurodevelopment.

**Figure 1:**
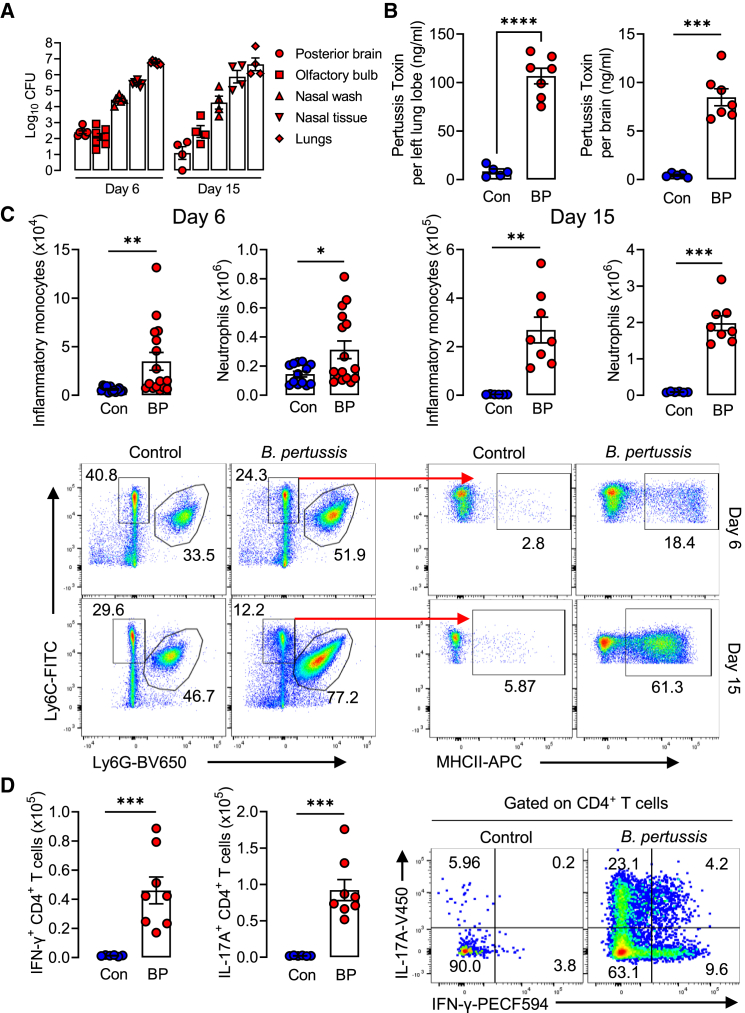
Local inflammation and dissemination of bacteria to the brain following respiratory challenge of neonatal mice with *B. pertussis* Ten-day old neonatal mice were aerosol challenged with live *B. pertussis*. (A) Colony forming units (CFU) in the lungs, nasal tissue, nasal wash, olfactory bulb, and posterior brain regions 6 and 15 days post-challenge with *B. pertussis*. (B) Concentration of PT by ELISA on lung and brain homogenates 15 days post-challenge with *B. pertussis.* (C) Number of inflammatory monocytes (MHCII^+^Ly6C^+^CD11b^+^) and neutrophils (Ly6c^+^Ly6G^+^) in the lungs with sample flow cytometry plots below. (D) Number of IFN-γ- or IL-17A-secreting CD4^+^ T cells in lungs at day 15 post aerosol challenge with *B. pertussis* by intracellular cytokine staining and flow cytometry, with sample flow plots to the right. Data are presented as mean ± SEM (*n* = 4–7 for CFU counts. *n* = 13–16 for Day 6 flow cytometry (controls: 5 male and 8 female, *B. pertussis*-infected: 8 male and 8 female) from two independent experiments. *n* = 6–8 for Day 15 FACS (Controls: 4 male and 2 female, *B. pertussis*-infected: 5 male and 3 female). (∗*p* < 0.05, ∗∗*p* < 0.01, ∗∗∗*p* < 0.001) by Student’s unpaired 2-tailed *t*-test.

Assessment of CFU counts 4 h post-B. *pertussis* aerosol challenge of 10-day-old neonatal mice revealed the infection of the lungs and nasal tract (Figure S1), which did not result in any fatalities. The CFU counts were less than 10^4^ CFU/lung, which is consistent with previous studies that this bacterial load is not lethal in neonatal mice, especially mice more than 7 days old.^31^ The CFU in the lungs and nose was substantially higher on day 6 and day 15 post-challenge (Figure 1A). Surprisingly, live bacteria were also detected and cultured from homogenates of the olfactory bulb and posterior brain regions of saline-perfused mice 6 and 15 days post aerosol challenge with *B. pertussis* (Figure 1A). Consistent with this, we found that PT was detectable by ELISA in the lung homogenates and at lower concentrations in the brain homogenates 15 days post-aerosol challenge with *B. pertussis* (Figure 1B). Infection of neonatal mice with *B. pertussis* resulted in the significant recruitment of inflammatory monocytes (MHCII^+^Ly6C^+^CD11b^+^) and neutrophils (Ly6G^+^CD11b^+^) into the lungs (Figure 1C) and nasal tissue (Figure S2A) when assessed 6 and 15 days post-challenge with *B. pertussis*. The numbers of CD4 T cells also significantly expanded in the lungs (Figure 1D) and nasal tissue (Figure S2B) of infected neonatal mice and these cells produced IL-17A and IFN-γ. We also detected *B. pertussis*-specific IL-17 and IFN-γ-secreting CD4 T cells in the lung when assessed 10 weeks after *B. pertussis* aerosol challenge of 10-day-old neonatal mice (Figure S3). These findings suggest that the *B. pertussis* infection of neonatal mice induces respiratory Th1 and Th17 cells, which are known to promote the infiltration and activation of inflammatory monocytes and neutrophils.

**Figure 2:**
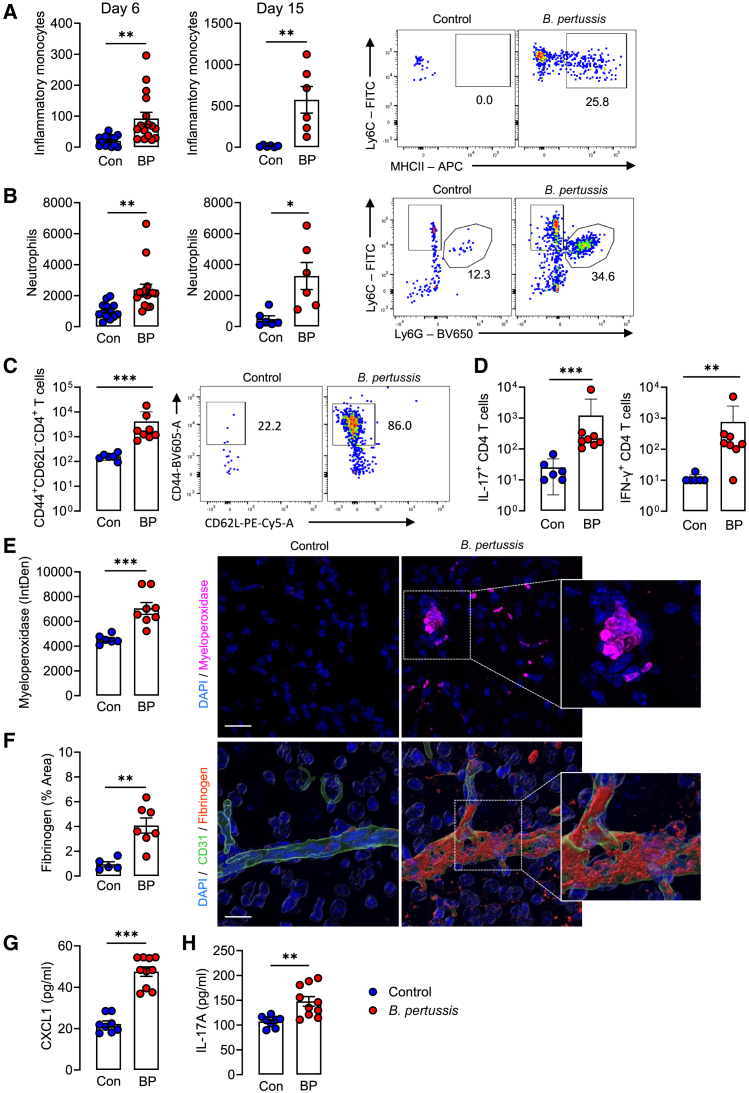
Neonatal infection with *B. pertussis* induces blood-brain barrier disruption and neuroinflammation Ten-day-old neonatal mice were aerosol challenged with live *B. pertussis*. (A and B) Flow cytometry analysis shows a mean number of inflammatory monocytes (MHCII^+^Ly6C^+^CD11b^+^) (A) and neutrophils (Ly6c^+^Ly6G^+^) (B) in the brain on days 6 and 15 post-challenge, with sample flow cytometry plots (day 15) to the right. (C) Number of activated T-cells (CD44^+^CD62L^−^CD4^+^) in the brain on day 15 post-challenge, with sample flow cytometry plots to the right. (D) Number of IFN-γ- or IL-17A-secreting CD4^+^ T cells in the brain by intracellular cytokine staining and flow cytometry. (E) Myeloperoxidase expression in the brain 6 days after neonatal infection, with representative confocal images to the right. Scale bar, 100 μm. (F) Fibrinogen deposition in the brain 6 days after neonatal infection, with 3D Imaris reconstructions to the right. Scale bar, 10 μm. (G and H) ELISA on brain homogenate showing mean CXCL1 (G) and IL-17A (H) concentrations 6 days post challenge with *B. pertussis.* Data are presented as mean ± SEM (*n* = 13–16 for Day 6 FACS (Controls: 5 male and 8 female, *B. pertussis*-infected: 8 male and 8 female) from two independent experiments). *n* = 6–8 for Day 15 FACS (Controls: 4 male and 2 female, *B. pertussis*-infected: 5 male and 3 female). *n* = 6–8 for confocal (Controls: 3 male and 3 female, *B. pertussis*-infected: 4 male and 4 female). n = 8–10 for ELISA (Controls: 3 male and 5 female, *B. pertussis*-infected: 8 male and 2 female). (∗*p* < 0.05, ∗∗*p* < 0.01, ∗∗∗*p* < 0.001) by Student’s unpaired 2-tailed t-test.

Flow cytometric analysis of immune cells in the CNS revealed a significant increase in the numbers of inflammatory monocytes (MHCII^+^Ly6C^+^CD11b^+^) and neutrophils (Ly6G^+^ CD11b^+^) in the brain of *B. pertussis*-infected mice compared with control mice at 6 and 15 days post-challenge (Figures 2A and 2B). Activated/memory CD44^+^CD62L^−^CD4^+^ T cells also infiltrated the brain of *B. pertussis*-infected mice (Figure 2C). Furthermore, these CD4 T cells produced IL-17A and IFN-γ; the numbers of IL-17A and IFN-γ-producing CD4 T cells were significantly higher in infected compared with control mice at 15 days (∗∗*p* < 0.01; ∗∗∗*p* < 0.001; Figure 2D). Confocal microscopy revealed that staining for myeloperoxidase (MPO), which is expressed by neutrophils and monocytes and has antimicrobial activity, was significantly higher in brain tissue of *B. pertussis*-infected, compared with control mice (∗∗∗*p* < 0.001; Figure 2E). The BP338 derivative of the Tohama 1 strain of *B. pertussis* used in this study secretes PT, which has previously been shown to disrupt the BBB.^32^^,^^33^ We, therefore, considered that the extravasation of immune cells into the brain may result from increased BBB permeability and/or chemotaxis of immune cells into the brain. We found an increased deposition of the high molecular weight plasma protein fibrinogen, an indicator of BBB disruption, in parenchymal brain sections from *B. pertussis*-infected, compared with control mice 6 days (∗∗*p* < 0.01; Figure 2F) and 15 days (Figure S4A) post-challenge. The chemokine CXCL1 is known to activate and recruit neutrophils into infected tissue. We showed that the concentration of CXCL1 was significantly increased in the brain 6 days (∗∗∗*p* < 0.001; Figure 2G) and 15 days (Figure S4B) post-respiratory challenge with *B. pertussis* (∗∗∗*p* < 0.001; Figure 2G). The concentration of IL-17A, which promotes expression of CXCL1 and thereby mobilizes blood-borne neutrophils to sites of infection,^34^ and has been associated with BBB disruption in multiple sclerosis,^35^ was also significantly increased in the brains of *B. pertussis*-infected mice 6 days (∗∗*p* < 0.01; Figure 2H) and 15 days (Figure S4C) post-challenge. Consistent with these findings, the expression of *Cxcl1* and *Il17a* mRNA was significantly increased in the hippocampus and cortex of mice 6 days post-challenge with *B. pertussis* (∗∗∗*p* < 0.001; Figures S5A and S5B). Additionally, mRNA expression of lipocalin-2, an antimicrobial peptide and constituent of neutrophil secondary granules, was significantly increased in the hippocampus and cortex of *B. pertussis*-infected mice (∗∗∗*p* < 0.001; Figure S5C).

These findings demonstrate that live bacteria disseminate to the brain of neonatal mice after aerosol challenge with *B. pertussis* and that this is associated with an inflammatory response in the brain and the respiratory tract. Activated Th1- and Th17-type CD4 T cells, inflammatory monocytes and neutrophils infiltrate the brain, perhaps because of increased BBB permeability and/or enhanced the production of the inflammatory cytokine, IL-17A, and the chemokine, CXCL1.

### Neonatal infection with *B. pertussis* triggers microglial activation

**Figure 3:**
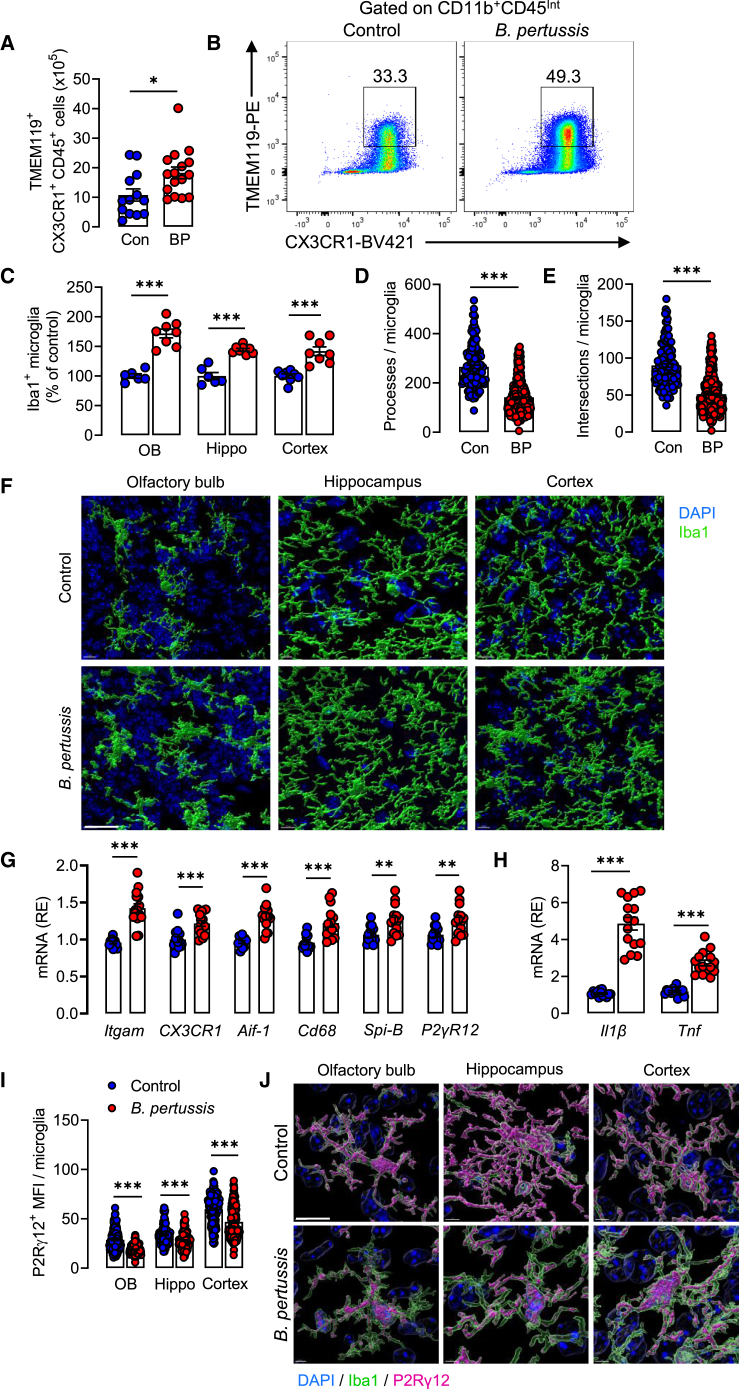
Neonatal infection with *B. pertussis* promotes microglial activation Ten-day old neonatal mice were aerosol challenged with live *B. pertussis*. (A) Number of microglia (TMEM119^+^CX3CR1^+^) in whole brain assessed by flow cytometry 6 days post challenge with *B. pertussis.* (B) Sample flow plots from control and *B. pertussis*-infected mice. (C) Number of microglia (Iba1^+^ with DAPI nuclear stain) in olfactory bulb, hippocampus and cortex by confocal microscopy 6 days post-challenge with live *B. pertussis*. (D) Number of processes per Iba1^+^cell. (E) Number of intersections per Iba1^+^ cell. (F) Representative confocal images and 3D Imaris reconstructions of microglia. Scale bar, 50 μm. (G) Relative mRNA expression of microglial activation markers (*Itgam*, *Cx3cr1*, *Aif-1*, *Cd68*, *Spi-B*, *P2γR12*). (H) Relative mRNA expression of *Il1b* and *Tnf* in hippocampus 6 days after neonatal challenge with *B. pertussis*. (I) P2γR12 mean fluorescence intensity (MFI) per microglia in olfactory bulb, hippocampus and cortex at 6 days post-infection. (J) Representative imaris reconstructions of Iba1^+^P2Rγ12^+^ microglia. Scale bar, 10 μm. Data are presented as mean ± SEM (*n* = 13–16 for FACS (Controls: 5 male and 8 female, *B. pertussis*-infected: 8 male and 8 female) from two independent experiments. n = 6–8 for Iba1^+^ cell counts and morphology (Controls: 3 male and 3 female, *B. pertussis*-infected: 4 male and 4 female). *n* = 13–15 for PCR analysis (Controls: 6 male and 7 female, *B. pertussis*-infected: 9 male and 6 female). *n* = 6 for P2γR12 analysis from one independent experiment (Controls: 3 male and 3 female, *B. pertussis*-infected: 4 male and 2 female). (∗*p* < 0.05, ∗∗*p* < 0.01, ∗∗∗*p* < 0.001) by Student’s unpaired 2-tailed *t*-test.

We have previously reported that the activation of microglia is associated with the infiltration of immune cells into the brain following *B. pertussis* infection in adult mice,^36^ and in aged mice^37^ or in a mouse model of Alzheimer’s disease.^38^ Therefore, we assessed microglial activation in response to neonatal infection with *B. pertussis*. Flow cytometric analysis indicated that there was an increase in the number of TMEM119^+^CX3CR1^+^CD45^Int^ microglia in the brain of infected mice 6 days post-challenge of neonates with *B. pertussis* (Figures 3A and 3B). We performed a multi-regional analysis of Iba1^+^ microglia by confocal microscopy to determine the spatial distribution of microglia. We found increased numbers of morphologically distinct microglia in the olfactory bulb (∗∗∗*p* < 0.001), hippocampus (∗∗∗*p* < 0.001), and cortex (∗∗∗*p* < 0.001) that exhibited fewer cell processes (∗∗∗*p* < 0.001) and intersections (∗∗∗*p* < 0.001), indicative of activated microglia (Figures 3C–3F). Furthermore, mRNA expression of *Itgam*, *Cx3cr1*, *Aif-1*, *Cd68*, *SpiB,* and *P2Rγ12,* reflective of microglial activation, were significantly increased in hippocampal tissue from *B. pertussis*-infected mice compared with control mice 6 days after challenge (∗∗∗*p* < 0.001; Figure 3G). These changes were accompanied by increased mRNA expression of the inflammatory cytokines *Il1b* and *Tnf* (∗∗∗*p* < 0.001; Figure 3H), and a decrease in expression of P2Rγ12 in microglia from the olfactory bulb, hippocampus, and cortex (∗∗∗*p* < 0.001; Figures 3I and 3J), indicating loss of homeostatic function. These findings demonstrate that microglia in *B. pertussis*-infected mice have an activated phenotype.

### Defective microglial phagocytosis and synaptic pruning in *B. pertussis*-infected mice

**Figure 4:**
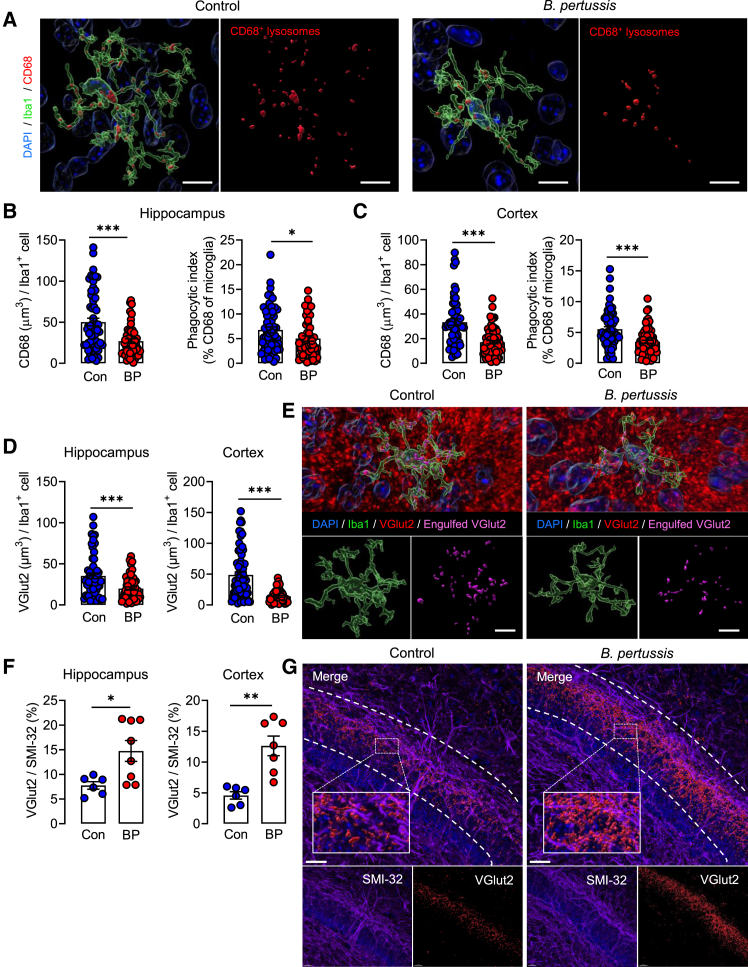
Impaired microglial phagocytic function and synaptic pruning of developing glutamatergic neurons in infected mice Ten-day old neonatal mice were aerosol challenged with live *B. pertussis*. (A) Representative Imaris reconstructions of CD68^+^ lysosomes inside Iba1^+^ microglia at day 6 post-challenge with live *B. pertussis*. Scale bars, 10 μm. (B) Single-cell volumetric analysis of lysosomes and microglial phagocytic index in hippocampus 6 days post-challenge with live *B. pertussis.* (C) Single-cell volumetric analysis of lysosomes and microglial phagocytic index in cortex 6 days post-challenge with live *B. pertussis.* (D) Pre-synaptic engulfment of VGlut2 by microglia in the hippocampus and cortex 6 days post-challenge with live *B. pertussis*. (E) Representative Imaris reconstructions of engulfed VGlut2 (pink) inside Iba1^+^ microglia (green). Scale bars, 10 μm. (F) VGlut2^+^ synaptic inputs normalised to SMI-32^+^ axonal volume in the hippocampus and cortex 6 days post-challenge with live *B. pertussis*. (G) Representative Imaris reconstructions of VGlut2^+^ synaptic inputs (red) co-localized with SMI-32^+^ axonal segments (magenta). Scale bars, 100 μm. Data are presented as mean ± SEM (*n* = 6–7 for CD68 and VGlut2 analysis from one independent experiment (Controls: 4 male and 3 female, *B. pertussis*-infected: 3 male and 3 female). *n* = 6–8 for SMI-32 analysis from one independent experiment (Controls: 4 male and 2 female. *B. pertussis*-infected: 4 male and 4 female). (∗*p* < 0.05, ∗∗*p* < 0.01, ∗∗∗*p* < 0.001) by Student’s unpaired 2-tailed *t*-test.

Microglial function, including phagocytosis, is impacted by microglial activation state.^38^ We assessed lysosomal volume as an indicator of phagocytic function 6 days after aerosol challenge of neonatal mice with *B. pertussis* and reported that CD68^+^ lysosomal volume was reduced in microglia from *B. pertussis*-infected animals (∗∗∗*p* < 0.001), as was the phagocytic index, in the hippocampus (∗*p* < 0.05) and cortex (∗∗∗*p* < 0.001; Figures 4A–4C).

We assessed the synaptic pruning of glutamatergic pre-synaptic inputs by analyzing VGlut2 engulfment by microglia. Our data show that VGlut2 engulfment per microglia was reduced in the hippocampus (∗∗∗*p* < 0.001) and cortex (∗∗∗*p* < 0.001) at day 6 post-challenge with *B. pertussis* (Figures 4D and 4E). Co-localization of VGlut2 with SMI-32, which labels neurofilaments, was used as a measure of synaptic inputs on cell bodies and dendrites. The data indicate that VGlut2-SMI-32 co-localization was increased in sections of the hippocampus and cortex prepared from *B. pertussis*-infected compared with control mice (∗*p* < 0.05; ∗∗∗*p* < 0.001; Figures 4F and 4G). The increase in number of synaptic inputs contacting SMI-32^+^ cell bodies/dendrites, together with the decreased VGlut2 engulfment, indicates that synaptic pruning by microglia was impaired by the infection of neonatal mice with *B. pertussis*.

### Neonatal infection with *B. pertussis* impairs neuronal circuit remodeling

**Figure 5:**
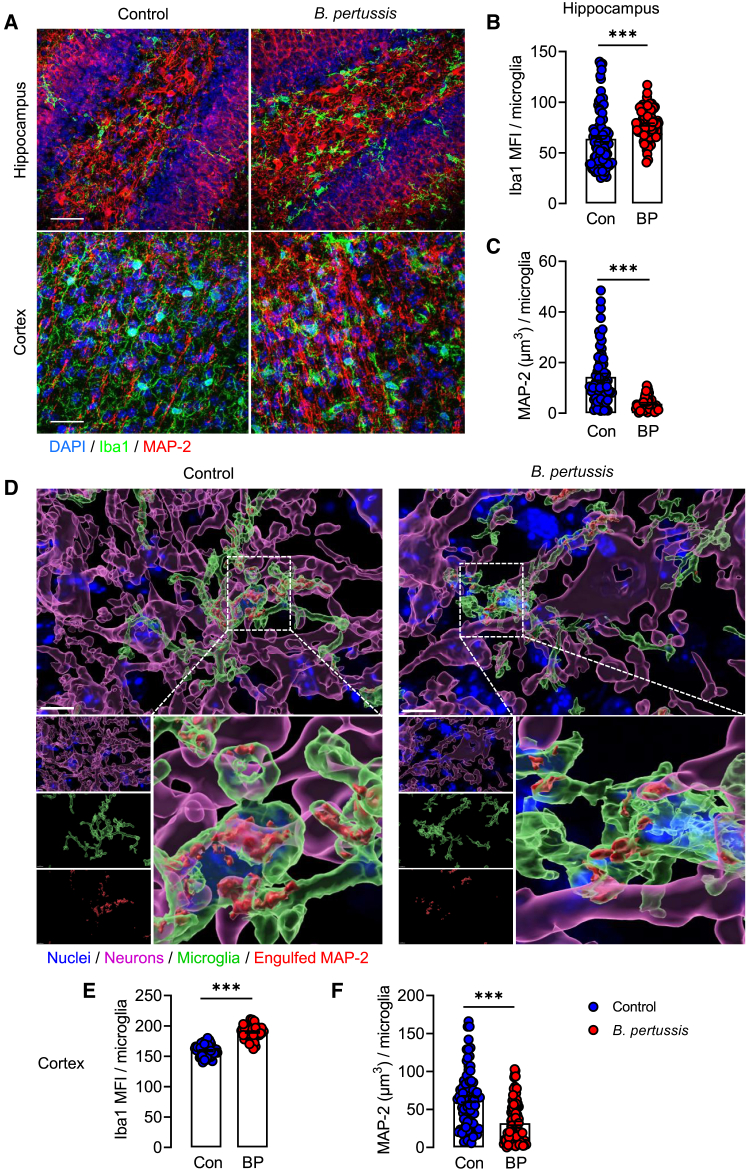
Defective neural circuit remodeling by microglia in infected mice Ten-day old neonatal mice were aerosol challenged with live *B. pertussis*. (A) Representative confocal z-stacks of Iba1 + MAP-2 immunofluorescence staining in hippocampus and cortex. Scale bars, 100 μm. (B) Iba1 staining intensity (MFI) per microglia in hippocampus 6 days post-challenge with *B. pertussis.* (C) MAP-2 engulfment per microglia in the hippocampus 6 days post-challenge with live *B. pertussis*. (D) Representative Imaris reconstructions for cell nuclei (blue) Iba1 (green), MAP-2^+^ dendrites and perikarya (magenta) and engulfed MAP-2 (red). Scale bars, 10 μm. (E) Quantification of Iba1 staining intensity (MFI) in cortex at 6 days post-challenge with live *B. pertussis*. (F) Quantification of MAP-2 engulfment by microglia in the cortex 6 days post-challenge with *B. pertussis*. Data are presented as mean ± SEM (*n* = 8–10 from one independent experiment (Controls: 4 male and 4 female, *B. pertussis*-infected: 6 male and 4 female). (∗∗∗*p* < 0.001) by Student’s unpaired 2-tailed *t*-test.

Early postnatal microglial sculpting of neuronal circuitry is critical for normal brain development.^39^ To evaluate whether the deficits in synaptic pruning in infected animals impacted neuronal circuit formation, we assessed the microglial engulfment of MAP-2, a neuron-specific cytoskeletal protein that is enriched in dendrites and perikarya. We show that Iba1^+^ fluorescent staining per microglial cell was upregulated in the hippocampus at day 6 post-challenge with B. pertussis and that the microglial engulfment of MAP-2 per cell was significantly reduced in the hippocampus (∗∗∗*p* < 0.001; Figures 5A–5C); similar changes were observed in the cortex (∗∗∗*p* < 0.001 Figures 5D–5F).

It has been consistently reported that ASD is linked with structural and functional differences in the cerebellum.^40^^,^^41^ Here we show that there was an increase in Iba1^+^ microglia in *B. pertussis*-infected mice and that these microglia had a lower number of phagocytic cups and reduced the engulfment of calbindin^+^ Purkinje cell dendrites (∗∗∗*p* < 0.001; ∗∗*p* < 0.01, ∗*p* < 0.05; Figures S6A–S6F). Significantly, the number of calbindin^+^ Purkinje cells per cerebellar lobule was reduced in *B. pertussis*-infected mice (∗*p* < 0.05; Figures S6G and S6H). These data indicate that neonatal infection with *B. pertussis* triggers an activated, inflammatory microglial phenotype that is less efficient at the postnatal remodeling of developing neuronal circuits.

### Neonatal infection with *B. pertussis* promotes long-term neuronal changes

**Figure 6:**
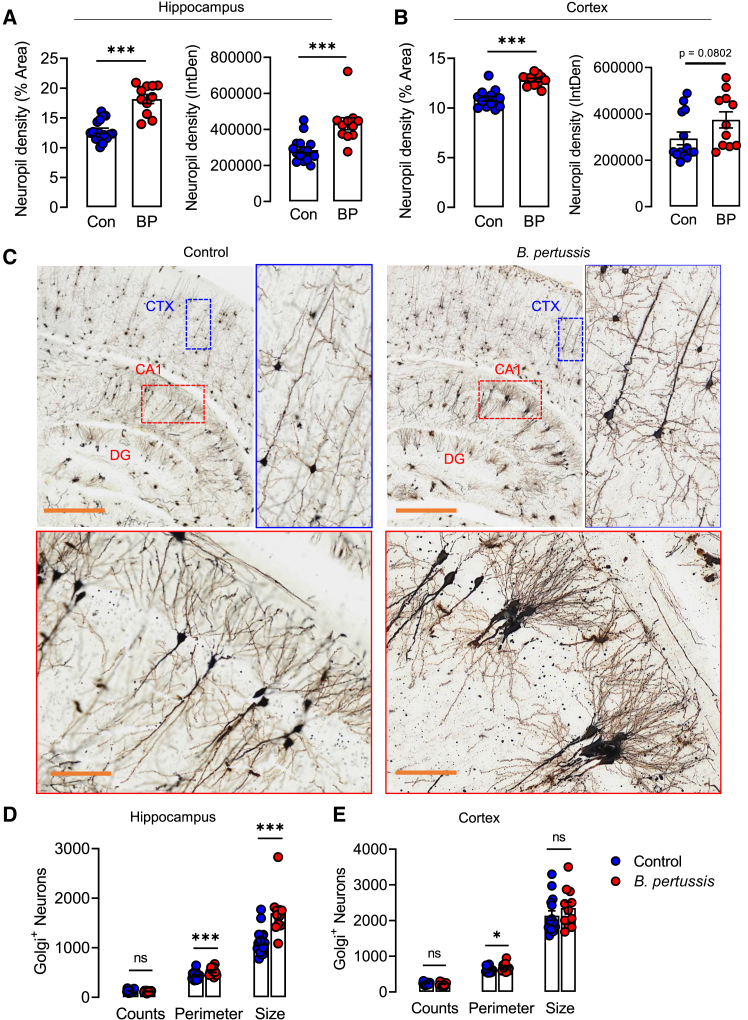
Neonatal infection with *B. pertussis* induces long-term neurodevelopmental alterations that persist into adulthood Ten-day old neonatal mice were aerosol challenged with live *B. pertussis*. (A) Neuropil density (% Area and Integrated density) in hippocampus ten weeks after neonatal infection with *B. pertussis.* (B) Neuropil density (% area and integrated density) in cortex ten weeks after neonatal infection with *B. pertussis*. (C) Representative Golgi-cox stained images from hippocampus and cortex. Scale bars, 100 μm and 10 μm for enlargements. (D) Quantification of neuronal cell body counts, neuronal perimeter and neuronal size in hippocampus. (E) Quantification of neuronal cell body counts, neuronal perimeter and neuronal size in cortex. Data are presented as mean ± SEM (*n* = 11–16 from two independent experiments (Controls: 11 male and 5 female, *B. pertussis*-infected: 8 male and 3 female). (∗*p* < 0.05, ∗∗∗*p* < 0.001) by Student’s unpaired 2-tailed *t*-test.

We conducted the Golgi-Cox staining of the adult brain at 10 weeks post *B. pertussis* challenge of neonatal mice to determine the long-term impact of the early postnatal defects in synaptic pruning and dendrite remodeling. Neuropil density of adult mice was increased in the hippocampus and cortex at 10 weeks post-challenge of neonatal mice with *B. pertussis* (∗∗∗*p* < 0.001; Figures 6A–6C). While there were no differences in the number of neuronal perikarya, the perimeter and size of pyramidal neurons in the stratum radiatum (CA1) and CA3 regions of the hippocampus were increased (∗∗∗*p* < 0.001; Figure 6D). Cell perimeter was also increased in the cortex (∗∗∗*p* < 0.001; Figure 6E). Our analysis indicates that the early postnatal neurodevelopmental changes that occur in response to neonatal infection with *B. pertussis* translate into white matter hyperplasia in adulthood.

### Neonatal infection with *B. pertussis* promotes autism-like behaviors in young adulthood

**Figure 7:**
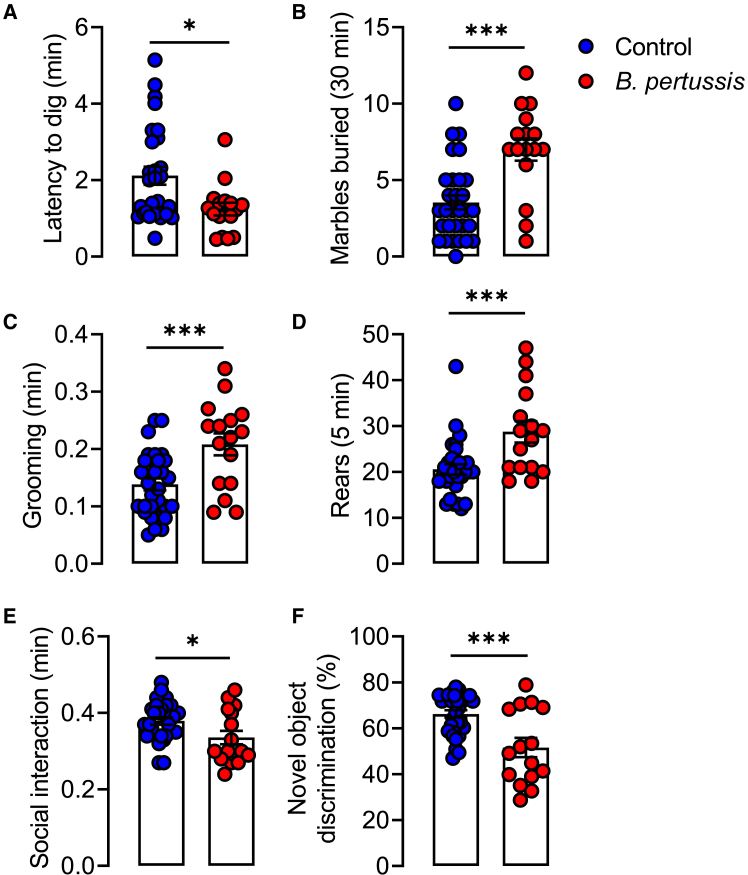
Neonatal infection with *B. pertussis* promotes ASD-related behavior in adult mice Neonatal mice (P10) were aerosol infected with live *B. pertussis* and stereotypic repetitive behavior, restricted interests and social interaction was assessed 8–10 weeks later in adulthood. (A) Latency to commence digging in marble burying test. (B) Number of marbles buried over 30 min in the marble burying test. (C) Duration of grooming over a 5 min test session. (D) Number of rears performed over a 5 min test session. (E) Duration of social interaction with a novel, age- and sex-matched mouse over a 5 min test session. (F) Novel object discrimination index in the novel-object recognition task. Data are presented as mean ± SEM (*n* = 16–30 from two independent experiments (Controls: 12 male and 18 female, *B. pertussis*-infected: 8 male and 8 female). (∗*p* < 0.05, ∗∗∗*p* < 0.001) by Student’s unpaired 2-tailed *t*-test.

It has been proposed that loss of synaptic refinement, such as that reported here, as well as gray and white matter cerebral lobe hyperplasia suggested by the changes identified with Golgi staining, are associated with behaviors that are characteristic features of ASD.^42^ We assessed marble burying as a measure of repetitive behaviors and found that the latency to dig was decreased and the number of marbles buried was increased, in mice 8–10 weeks post neonatal challenge with *B. pertussis* (∗*p* < 0.05, ∗∗∗*p* < 0.001; Figures 7A and 7B). Mice were assessed in the open field to examine exploratory behavior and stereotypic, repetitive activity. Exploratory behavior, assessed by the number of squares crossed, was unaffected by neonatal *B. pertussis* infection (Figure S7A). However, the time spent grooming and the number of rears, which are measures of repetitive behavior, was greater in mice that had been infected with *B. pertussis* (∗∗∗*p* < 0.001; Figures 7C and 7D). Conversely, the duration of social interaction with a novel age- and sex-matched mouse was reduced in *B. pertussis*-infected animals (∗*p* < 0.05; Figure 7E). In the novel object recognition task, there were no differences in total time spent exploring the familiar and novel objects (Figure S7B), but the novel object discrimination index was reduced in response to *B. pertussis* infection (∗∗∗*p* < 0.001; (Figure 7F). We stratified the behavioral data according to sex and observed greater ASD-like behavioral differences in *B. pertussis*-infected and controls, in male compared with female, mice (Figure S8). We also assessed the neurological/behavioral effect of *B. pertussis* infection in adult mice. Eight-week-old mice were aerosol infected with *B. pertussis* and behaviors that are characteristic features of ASD were assessed at 8 weeks post-aerosol infection with *B. pertussis.* The data indicate that the respiratory infection of adult mice with *B. pertussis* does not alter grooming, rearing, social interaction, or recognition memory (Figures S9A–S9D). Collectively, these data indicate that neonatal respiratory challenge with *B. pertussis* drives abnormal brain development by impairing postnatal microglial function, culminating in the onset of ASD-like behaviors in adulthood, especially in male mice.

## Discussion

We demonstrate that the infection of neonatal mice with the respiratory pathogen *B. pertussis* results in neuroinflammation and abnormal neurodevelopment, culminating in ASD-like behavior in young adulthood. We found that inflammatory responses during a critical period in neurodevelopment impaired synaptic pruning and neuronal circuit remodeling by microglia and these changes were associated with invasion of *B. pertussis* into the brain. These early postnatal impairments in microglial function were associated with persistent deficits in neurodevelopment, which, we propose, resulted in observed ASD-like related behaviors in young adulthood.

Neuroinvasion of respiratory pathogens and resulting neuroinflammation and encephalitis are well documented for viruses. SARS-CoV-2 has been found in the olfactory bulb, resulting in disrupted vascular homeostasis, neutrophil infiltration, and microglial activation, with an increased risk of neurological disorders.^43^^,^^44^ Respiratory bacteria, including *Haemophilus influenzae type b* and *Neisseria meningitidis,* can disseminate to the brain and cause meningitis or encephalitis.^45^ Although it is considered that *B. pertussis* is confined to the respiratory tract, bacteria have been detected in the cerebrospinal fluid of human infants with severe pertussis^46^ and in the blood, liver, and spleen of neonatal mice, where bacterial dissemination was linked with defective IFN-γ-secreting NK cells.^30^ Furthermore, we have cultured live bacteria from livers of immunodeficient IFN-γR^−/−^ adult mice infected with *B. pertussis*.^29^ These studies, together with our demonstration that IL-17 producing T cells play a central role in the elimination of *B. pertussis* from the nasal cavity,^47^ suggest that NK cells, Th1, and Th17 cells are crucial for controlling local infection, but may also prevent the dissemination of bacteria outside of the respiratory tract. In the present study, we found that Th1 and Th17 responses were induced following the infection of neonatal mice with *B. pertussis* and that these T cells were recruited to the respiratory tract and also migrated to the brain.

Interestingly, maternal immune activation, induced by poly(I:C), resulted in the development of ASD-like behavior in offspring with evidence pointing to a role for IL-17 and Th17 cells.^48^ Furthermore, IL-17 production is elevated in children with ASD^49^; however, its role in the development of ASD is unclear. We have reported that IL-17 plays a pathogenic role in experimental autoimmune encephalomyelitis, a mouse model of multiple sclerosis, promoting the recruitment of neutrophils and inflammatory monocytes to the CNS via CXCL1 production.^50^ Here we found increased expression of IL-17A and CXCL1 in the brain, as well as the recruitment of Th17 cells, neutrophils, and inflammatory monocytes to the brains of mice infected with *B. pertussis* at a critical period in early postnatal neurodevelopment. Immune cell recruitment to the brain was associated with enhanced BBB permeability. As noted previously, the *B. pertussis* virulence factor, PT, can reduce BBB integrity by altering the organizational structure and permeability of brain microvascular endothelial cells.^33^

In addition to the recruitment of activated immune cells into the CNS, we found marked activation of microglia in the olfactory bulb, hippocampus, cortex, and cerebellum of infected mice. We observed a morphological shift from a quiescent, ramified state to a morphology with retracted processes and enlarged cell soma, along with an upregulation in activation markers, indicative of an activated, inflammatory microglial phenotype. Furthermore, microglial expression of P2Rγ12 was reduced, suggesting a shift from homeostasis.^51^ Interestingly, it has been shown that microglia with deficits in P2Rγ12-dependant purinergic signaling in the hippocampus of the neuroligin-4 knockout mouse model of ASD have reduced phagocytic function.^52^ Notably, PT induces microglial activation, and in co-culture experiments, PT inhibition of Gi proteins led to elevated cAMP and reduced microglial branching, hallmarks of microglial activation.^53^

Microglia are critical for normal brain development and play a vital role in synaptic pruning, which is essential for neuronal circuit assembly and maturation.^54^^,^^55^ We found infection-induced changes in microglial activity impacted the ability of the cells to prune synaptic connections and sculpt neuronal circuitry. Microglia from animals infected with *B. pertussis* exhibited reduced lysosomal volume, became less phagocytic and engulfed less synaptic material from developing glutamatergic neurons in the hippocampus and cortex. Our demonstration of the early postnatal defective pruning of glutamatergic pre-synaptic inputs and altered dendrite remodeling of pyramidal neurons, collectively indicate abnormal neuronal maturation.

Purkinje cells in the cerebellum play a critical role in regulating social behaviors and altered functional cerebellar connectivity and Purkinje cell pathology is consistently reported in both mouse models of ASD and in human individuals with ASD.^56^^,^^57^ Here we show early postnatal reductions in Purkinje cell numbers in the cerebellum of infected mice, a neuropathological feature of human ASD.^58^ This was associated with the accumulation of microglia with reduced phagocytic cups in the developing cerebellum and reduced engulfment of calbindin. Our findings provide further evidence of deficiencies in microglia-dependent developmental phagocytosis and postnatal sculpting and refinement of neuronal populations linked to ASD phenotypes in response to neonatal infection with *B. pertussis*.

The imbalance between excitatory and inhibitory neuronal circuit formation during a critical period in development is a feature of ASD that can lead to hyper-excitability and permanent alterations in neuronal connectivity and function.^59^^,^^60^ Here we found increased neuropil density, particularly in CA1 hippocampal pyramidal neurons and in layer V cortical pyramidal neurons, in adult mice that were infected with *B. pertussis* as neonates. This indicates long-term enhancement of local excitatory connectivity and draws a striking parallel with reports of increased spine density with reduced developmental spine pruning of pyramidal neurons in the temporal lobe of postmortem samples from individuals with ASD.^61^ These findings are also consistent with observations that children with ASD are at higher risk for epilepsy, often the result of a hyper-excitable CNS state,^62^ that children with a history of pertussis have a higher rate of seizures,^63^ and that PT increases excitatory neuronal glutamate release,^64^ while also inhibiting the inhibitory neurotransmitter GABA.^65^

Defective synaptic refinement and early gray and white matter cerebral lobe hyperplasia in autistic children is associated with repetitive behaviors and reduced sociability.^42^^,^^66^ We show that white matter hyperplasia, as suggested by enhanced Golgi^+^ neuropil density, was associated with stereotypic repetitive behaviors such as excessive rearing, grooming, and marble burying, as well as deficits in social interaction and impairments in recognition memory in adult mice 10 weeks post neonatal challenge with *B. pertussis*. We propose that ASD-related behaviors in older mice result from neurodevelopmental changes in neonatal mice following respiratory infection with *B. pertussis*. These changes include deficits in microglial function, the presence of live *B. pertussis* in the CNS, and potentially the activity of PT and dermonecrotic toxin. Mechanistically, these behavioral changes are consistent with the demonstration that mice with excess excitatory neurons in the neocortex exhibit autism-like behavior.^67^

Our findings demonstrate that the respiratory pathogen *B. pertussis* is neuroinvasive in neonatal mice and that postnatal neuroinflammation and disruption in microglial function during a critical period of neurodevelopment can lead to pathology consistent with an ASD-like phenotype. These data provide a biological explanation for the epidemiological observations of corresponding *B. pertussis* and ASD rates in Sweden and the US, and the development of selective communication deficits in infants after severe pertussis disease. Our findings demonstrate the neurological consequences of severe respiratory infection with *B. pertussis* in neonatal mice and highlight a potential increased risk of ASD following *B. pertussis* infection during infancy in humans.

### Limitations of the study

Our study has some limitations. It did not examine different challenge doses of *B. pertussis* used to infect the neonatal mice, the persistence of the behavioral changes beyond 10 weeks post *B. pertussis* challenge, or the role of PT in the observed behavioral phenotype. These are aspects that may be addressed in future studies. Furthermore, the study was performed in a mouse model that is not considered a model of whooping cough disease. However, the mouse respiratory challenge model closely mimics the effects of *B. pertussis* infection and host immune responses in humans. Future studies that examine the impact of childhood pertussis on the development of ASD in humans are warranted.

## Resource availability

### Lead contact

Requests for further information and resources should be directed to and will be fulfilled by the lead contact, Prof Kingston H. G. Mills (kingston.mills@tcd.ie).

### Materials availability

This study did not generate new unique reagents.

### Data and code availability

• The data have been deposited at DRYAD and will be publicly available as of the date of publication at https://doi.org/10.5061/dryad.vq83bk43q.
• This article does not report the original code.
• Any additional information required to reanalyze the data reported in this article is available from the lead contact upon request.

## Acknowledgments

This work was supported by a research grant from ILiAD technologies to M.A.L. and K.H.G.M. and from Science Foundation Ireland / Taighde Éireann – Research Ireland under Grants number 16/IA/4468 and 22/FFP-A/10297 to K.H.G.M.

## Author contributions

Study design: K.H.G.M., M.A.L., S.G., K.R., and E.O.N.; supervision and funding acquisition: K.H.G.M. and M.A.L.; methodology, data collection and visualization: E.O.N., L.C., C.N.C., S.O.B., G.M.M., and B.M.; writing, review and editing article: E.O.N., K.H.G.M., M.A.L, S.G. and K.R.

## Declaration of interests

Kingston Mills is an inventor on a patent around an adjuvant for a pertussis vaccine and has received research funding and acted as a consultant for vaccine manufacturers.

Keith Rubin and Steven Glazer are employed by ILiAD Biotechnologies, which is developing a vaccine for the prevention of pertussis.

## STAR★Methods

### Key resources table

**Table:** 

REAGENT or RESOURCE	SOURCE	IDENTIFIER
**Antibodies**		

Anti-mouse CD103 - BV786 (1:200)	BD Biosciences	Clone: M290; RRID: AB_2738744
Anti-mouse CD11b APCeFluor780 (1:500)	Invitrogen	Clone: M1/70; RRID: AB_1603193
Anti-mouse CD11c - PE/Cyanine5 (1:400)	BioLegend	Clone: N418; RRID:AB_493566
Anti-mouse CD3 - BV650 (1:200)	BioLegend	Clone: 17A2; RRID: AB_11204249
Anti-mouse CD4 - APCeFluor780 (1:200)	eBioscience	Clone: RM4-5; RRID: AB_1272183
Anti-mouse CD44 - BV605 (1:400)	BioLegend	Clone: IM7; RRID: AB_2562451
Anti-mouse CD45 – PE (1:400)	eBioscience	Clone: 30-F11; RRID:AB_465669
Anti-mouse CD62L - PE/Cyanine7 (1:200)	BioLegend	Clone: MEL-14; RRID: AB_313103
Anti-mouse CD69 – APC (1:200)	BioLegend	Clone: H1.2F3; RRID: AB_492843
Anti-mouse CD8a - Alexa Fluor 700 (1:200)	eBioscience	Clone: 53-6.7; RRID: AB_494005
Anti-mouse CX3CR1 – BV711 (1:200)	BioLegend	Clone: SA011F11; RRID: AB_2565939
Anti-mouse IFNγ - BV711 (1:200)	BioLegend	Clone: XMG1.2; RRID: AB_11219588
Anti-mouse IL-17A – FITC (1:200)	BioLegend	Clone: TC11-18H10.1; RRID: AB_536010
Anti-mouse IL-17A - V450 (1:200)	BD Biosciences	Clone: TC11-18H10; RRID: AB_1727540
Anti-mouse Ly6C – FITC (1:400)	BD Biosciences	Clone: AL-21 RRID:AB_394628
Anti-mouse Ly6G - BV650 (1:400)	BioLegend	Clone: 1A8; RRID: AB_2565881
Anti-mouse MHCII – PE (1:400)	Thermo Fisher Scientific	Clone: M5/114.15.2; RRID: AB_465928
Anti-mouse Tmem-119 – PE (1:400)	Thermo Fisher Scientific	Clone: V3RT1GOsz; RRID: AB_2848262
Chicken polyclonal to Anti-Neurofilament heavy polypeptide (1:1000)	Abcam	CAT# ab4680; RRID: AB_304560
Chicken polyclonal to MAP2 (1:1000)	Abcam	CAT# ab5392; RRID: AB_2138153
Goat polyclonal to IBA1 (1:500)	Abcam	CAT# ab5076; RRID: AB_2224402
Rabbit monoclonal to Calbindin (1:1000)	Abcam	CAT# ab229915; RRID: AB_3086776
Rabbit monoclonal to IBA1 (1:1000)	Wako Chemicals	CAT# 019–19741; RRID: AB_839504
Rabbit monoclonal to Myeloperoxidase (1:500)	Abcam	CAT# ab221847; RRID: AB_3086778
Rabbit monoclonal to VGLUT2 (1:1000)	Abcam	CAT# ab216463; RRID: AB_2893024
Rabbit polyclonal to CD68 (1:250)	Abcam	CAT# ab125212; RRID: AB_10975465
Rabbit polyclonal to Fibrinogen (1:50)	Abcam	CAT# ab34269; RRID: AB_732367
Rat monoclonal to CD31 (1:50)	Abcam	CAT# ab7388; RRID: AB_305905
Rat monoclonal to P2RY12 (1:100)	BioLegend	CAT# 848002; RRID: AB_2650633
Bordetella pertussis Toxin Monoclonal Antibody (1280/204)	Thermo Fisher Scientific	CAT# MA1-83016; RRID: AB_927192
Rabbit Bordetella pertussis Pertussis toxin subunit 1 Polyclonal Antibody	MyBioSource	CAT# MBS1491326; RRID: AB_3251497
Donkey anti-rabbit IgG Secondary antibody -AlexaFluor 647 (1:1000)	Invitrogen	CAT# A31573; RRID: AB_2536183
Goat anti-chicken IgG Secondary antibody – Alexa Fluor 488 (1:1000)	Invitrogen	CAT# A11039; RRID: AB_142924
Goat anti-chicken IgG Secondary antibody – Alexa Fluor 633 (1:1000)	Invitrogen	CAT# A21103; RRID: AB_1500591
Goat anti-Rabbit IgG Secondary Antibody - Alexa Fluor 488 (1:1000)	Invitrogen	CAT# A11034; RRID: AB_2576217
Donkey Anti-Rabbit IgG Secondary antibody - Alexa Fluor 594 (1:1000)	Invitrogen	CAT# A21207; RRID: AB_141637
Goat anti-Rabbit IgG Secondary Antibody - Alexa Fluor 488 (1:1000)	Invitrogen	CAT# A11034; RRID: AB_2576217
Goat anti-Rat IgG Secondary Antibody - Alexa Fluor 488 (1:1000)	Invitrogen	CAT# A11006; RRID: AB_2534074
Goat anti-Rat IgG Secondary Antibody, Alexa Fluor 633	Invitrogen	CAT# A21094; RRID: AB_2535749

**Bacterial and virus strains**		

*Bordetella pertussis* (BP338) *nal-1* derivative of Tohama-1	NIBSC	N/A

**Chemicals, peptides, and recombinant proteins**		

PMA	Sigma-Aldrich	Cat#: P1585
Ionomycin	Sigma-Aldrich	Cat#: I0643
Brefeldin A	Sigma-Aldrich	Cat#: B7651
Purified Rat Anti-Mouse CD16/CD32 (Mouse BD Fc Block™)	BD Pharmingen™	Cat#: 553141
ACK Lysing Buffer	ThermoFisher	Cat#: A1049201
Mouse ACTB (Actin, Beta) Endogenous Control (VIC™/MGB probe, primer limited)	ThermoFisher	Cat#: 4352341E
PBS Tablets	Gibco	Cat#: 18912-014
2-Methylbutane	Sigma-Aldrich	Cat#: 59070
Ethylene glycol	Sigma-Aldrich	Cat#: 324558
Triton™ X-100	Sigma-Aldrich	Cat#: T8787
Normal Goat Serum	abcam	Cat#: ab7481
Normal Horse Serum	abcam	Cat#: ab139501
Mounting Medium With DAPI - Aqueous, Fluoroshield	abcam	Cat#: ab104139

**Critical commercial assays**		

NucleoSpin RNA, Mini kit for RNA purification	Macherey-Nagel	Cat#: 740955.50
High capacity cDNA reverse transcription kit	Applied Biosystems, Biosciences	Cat#: 4368814
Mouse ACTB (Actin, Beta) Endogenous Control (VIC™/MGB probe, primer limited)	Applied Biosystems, Biosciences	Cat#: 4352341E
LIVE/DEAD™ Fixable Aqua Dead Cell Stain Kit	Life Technologies	Cat#: L34957
CountBright™ Absolute Counting Beads	Life Technologies	Cat#: C36950
Foxp3 Transcription Factor staining buffer set	eBioscience	Cat#: 00-5523-00
Mouse CXCL1/KC DuoSet ELISA	RandD	Catalog #: DY453
ELISA MAX™ Standard Set Mouse IL-17A	BioLegend	Catalog #: 432501
FD Rapid GolgiStain™ Kit (Large)	FD NeuroTechnologies, INC.	Catalog #: PK401

**Deposited data**		

Raw data	This paper	Data base: DRYAD DOI: https://doi.org/10.5061/dryad.vq83bk43q

**Experimental models: Organisms/strains**		

Mouse; C57BL/6J	Comparative Medicine Unit, Trinity College Dublin	N/A

**Oligonucleotides**		

*Il1b* (Mm00434228_m1)	Applied Biosystems	Cat#: 4331182
*Il17a* (Mm00439618_m1)	Applied Biosystems	Cat#: 4331182
*Tnf* (Mm00443258_m1)	Applied Biosystems	Cat#: 4331182
*Cxcl1* (Mm04207460_m1)	Applied Biosystems	Cat#: 4331182
*Aif1* (Mm00479862_g1)	Applied Biosystems	Cat#: 4331182
*Cx3cr1* (Mm02620111_s1)	Applied Biosystems	Cat#: 4331182
*Itgam* (Mm00434455_m1)	Applied Biosystems	Cat#: 4331182
*Spib* (Mm03048233_m1)	Applied Biosystems	Cat#: 4331182
*Tmem119* (Mm00525305_m1)	Applied Biosystems	Cat#: 4331182
*Cd68* (Mm03047343_m1)	Applied Biosystems	Cat#: 4331182
*P2rγ12* (Mm01950543_s1)	Applied Biosystems	Cat#: 4331182
*Lcn2* (Mm01324470_m1)	Applied Biosystems	Cat#: 4331182

**Software and algorithms**		

Prism 9	https://www.graphpad.com/features	RRID:SCR_002798
FlowJo v10.9.0	https://www.flowjo.com/solutions/flowjo	RRID:SCR_008520
Cytek SpectroFlo	https://cytekbio.com/pages/spectro-flo	RRID:SCR_025494
BD FACSDiva Software	http://www.bdbiosciences.com/instruments/software/facsdiva/index.jsp	RRID:SCR_001456
FiJi ImageJ	https://imagej.net/software/fiji/	RRID:SCR_002285
Imaris	http://www.bitplane.com/imaris/imaris	RRID:SCR_007370

### Experimental model and study participant details

#### Mice

C57BL/6J mice were bred in-house from established colonies. C57BL/6J male and female adult (8 weeks at beginning of experiment) and neonatal (10-day old at beginning of experiment) mice were housed in a pathogen-free facility in the Comparative Medicine Unit, Trinity College Dublin. All animal experiments were conducted in accordance with European Union regulations under project licence (AE19136/P117) authorized by the Irish Health Products Regulatory Authority and with local ethical approval (Animal Research Ethics Committee, Trinity College Dublin; Reference number: 060619).

### Method details

#### Respiratory challenge with *B. pertussis*

Ten-day old neonatal mice or 8 week old adult were infected by aerosol challenge with *B. pertussis* (Bp338 strain) from a culture of 1 x 10^9^ CFU/mL using a Nebulizer (PARI TurboBOY SX) for 10 min as previously described.^47^ Live bacteria were enumerated in homogenized/digested lung, nasal tissue, olfactory bulb, and the remaining brain and in nasal wash by performing CFU counts using Bordet-Gengou agar plates for *B. pertussis*.

#### Flow cytometry analysis of cells from nasal tissue, lungs and brain

Nasal tissue and lung were digested with collagenase-D (1 mg/ml; Roche) and DNase I (10 mg/mL; Sigma-Aldrich) for 1 h at 37°C with agitation. Nasal tissue and lung were passed through a 70 μm cell strainer to acquire a single cell suspension. For preparation of immune cells in brain, mice were sacrificed and perfused with 20 mL of ice-cold PBS (pH 7.4) before removal of the brain. The brains were collected in cRPMI and chemically digested using collagenase-D (1 mg/ml; Roche) and DNase I (10 mg/mL; Sigma-Aldrich) for 1 h at 37°C with agitation. Brain tissue homogenates were resuspended in 40% isotonic Percoll and layered over 70% isotonic Percoll before centrifugation (1600 rpm for 20 min). Mononuclear cells were removed from the interface of the Percoll gradients, passed through a 70 μm filter and washed in cRPMI. Ammonium chloride-potassium (ACK) buffer was used to lyse red blood cells. The cells were washed in PBS and incubated in LIVE/DEAD Aqua (1:600; Invitrogen) followed by Fcγ block (αCD16/CD32 FcγRIII; 1:200; BD Biosciences) to block IgG Fc receptors. Cells were then washed and surface-stained with antibodies specific for CD11b (clone: M1/70), Ly-6C (AL-21), MHC-II (M5/114.15.2), Ly-6G (1A8), CD11c (N418). For intracellular cytokine staining, cells were stimulated with PMA (50 ng/mL; Sigma) and ionomycin (500 ng/mL; Sigma) in the presence of brefeldin A (5 μg/mL; Sigma) for 4 h at 37°C. Cells were fixed and permeabilized using the eBioscience Foxp3/Transcription Factor Staining Buffer Kit according to the manufacturer’s protocol (Thermo Fisher Scientific). Cells were stained intracellularly with antibodies specific for IL-17A (clone: TC11-18H10) and IFN-γ (XMG1.2). Microglia were analyzed by staining brain mononuclear cells with CX3CR1 (clone: SA011F11) and TMEM119 (clone: V3RT1GOsz). Fluorescence minus one (FMO) controls were used. Flow cytometric analysis was performed on an LSRFortessa or a Cytek Aurora cytometer. Data were acquired using Diva software (BD Biosciences) or SpectroFlo software (Cytek Biosciences) respectively, and analyzed using FlowJo v10.9.0 software (BD Biosciences). The flow cytometry gating strategies are shown in Figures S10 and S11.

#### ELISA

Brains were removed from perfused euthanized mice and mechanically homogenised using a tissue lyser (QIAGEN). The concentrations of CXCL1 (RandD Systems; DY453) and IL-17A (BioLegend; 432501) were quantified in brain homogenate supernatants by ELISA according to the manufacturer’s instructions and read using a SpectraMax ABS microplate reader (Molecular Devices). The concentration of PT in supernatant derived from the left lung lobe and whole brain was quantified via ELISA using a capture anti-PT mouse monoclonal antibody (MA1-83016) and a biotin-conjugated anti-PT subunit 1 rabbit polyclonal antibody (MBS1491326) for detection.

#### RT-qPCR

mRNA expression of inflammatory cytokines and microglial markers was assessed in sample homogenates from the olfactory bulb, hippocampus and cortex. RNA was isolated using Nucleospin RNAII Kit (Macherey-Nagel, Duren, Germany) and cDNA was prepared using a High-Capacity cDNA RT kit according to the manufacturer’s instructions (Applied Biosystems, UK). Real-time PCR was performed with predesigned Taqman gene expression assays using an Applied Biosystems 7500 Fast Real-Time PCR machine (Applied Biosystems, Germany). Samples were assayed as previously described,^68^ with β-actin (Mm00407939_s1) as the endogenous control to normalize gene expression data. Gene expression was calculated relative to the endogenous control samples and to the control sample giving an RQ value (2^- DDCt^), where Ct is the threshold cycle).

#### Tissue preparation and confocal immunofluorescence microscopy

Animals were deeply anesthetized with sodium pentobarbital and transcardially perfused with 0.89% sterile saline and fixed with 4% paraformaldehyde in 0.1 M phosphate buffered saline (pH 7.4). Brains were post-fixed for 24 h and transferred to 30% sucrose for 72 h for cryoprotection. 30 μm sections were cut using a cryostat and free-floating sections were stored in freezing solution at −80°C in preparation for immunohistochemistry. Sections were permeabilized using 0.3% Triton X-100 in PBS and blocked using either 10% normal goat serum or 10% normal horse serum in PBS. Sections were incubated in primary antibody solution (e.g., Myeloperoxidase, CD31 + Fibrinogen, Iba1 + P2Rγ12, Iba1 + CD68, Iba1 + VGlut2, SMI-32 + VGlut2, Iba1 + MAP-2 or Iba1 + Calbindin) containing 1% BSA overnight at 4°C. Sections were washed in PBS before incubation in secondary antibodies conjugated to AlexaFluor fluorochromes containing 1% BSA in PBS for 2 h in the dark (e.g., anti-rabbit 488 for Myeloperoxidase, anti-rat 488 and anti-rabbit 647 for CD31 + Fibrinogen, anti-goat 488 and anti-rat 633 for Iba1 + P2Rγ12, anti-goat 488 and anti-rabbit 594 for Iba1 + CD68, anti-goat 488 and anti-rabbit 546 for Iba1 + VGlut2, anti-rabbit 488 and anti-chicken 633 for VGlut2 + SMI-32, anti-goat 488 and anti-chicken 633 for MAP-2 + Iba1). Sections were washed in PBS, counterstained with DAPI and mounted onto gelatin-coated slides (mounting media with DAPI [Abcam]). Rabbit polyclonal antibodies used in the study were tested for and shown not to have reactivity with *B. pertussis*.

#### Image acquisition and analysis

Images were acquired using a Leica SP8 confocal microscope (x40 and ×63 objective lens with oil immersion). 70–85 z stack planes were taken with 0.35 μm spacing and images were processed on the LAS X Life Science Microscope Software Platform. Fibrinogen deposition and Myeloperoxidase expression was quantified as an assessment of blood brain barrier integrity using FIJI. Morphometric cell counting of microglia from the olfactory bulb, hippocampus, cortex and cerebellum were quantified using the ‘’Cell-Counter’’ plug-in on FIJI. Morphological assessment of microglial complexity was performed using the ‘’Analyze Skeleton’’ plug-in on FIJI.

#### Microglial engulfment and phagocytosis analysis

Free-floating sections (30 μm) were immunostained with Iba1 and CD68, Iba1 and VGlut2 or Iba1 and MAP-2 to assess lysosomal volume, synaptic pruning or dendrite remodeling by microglia respectively. For each mouse, between 6 and 9 regions of interest within the suprapyramidal region at the level of the stratum radiatum (CA1), CA3 associational connections and the dentate gyrus (DG) within the hippocampus and areas of the association cortex, primary somatosensory cortex and piriform cortex were considered for analysis. Raw images were acquired under a 63× oil objective on a confocal microscope and processed on Imaris (Bitplane). For assessment of synaptic pruning, after background subtraction using median and Gaussian filters, Iba1^+^ cells and VGlut2^+^ presynaptic inputs were surface rendered with 0.2 μm and 0.1 μm smoothing, respectively. A mask was applied within Iba1^+^ reconstructed microglia for VGlut2 and the volume of engulfed VGlut2 within microglia was measured. Similarly, for assessment of dendrite remodeling, a mask was applied within Iba1^+^ reconstructed microglia for MAP-2 and the volume of engulfed MAP-2 within microglia was measured. For assessment of lysosomal volume, Iba1^+^ cells and CD68^+^ lysosomes were surface rendered, a mask was applied within Iba1^+^ reconstructed microglia for CD68, and the volume of CD68^+^ lysosomes per microglia was calculated. The phagocytic index of microglia was calculated using the following formula: volume of CD68^+^ lysosomes/volume of Iba1^+^ microglia x 100.

#### Golgi-Cox staining

Sagittal sections (50 μm) of mouse brain were cut using a cryostat and stained using the Golgi-cox method (FD Rapid GolgiStain Kit) according to the manufacturer’s instructions (FD NeuroTechnologies, INC). Images were taken using an SLIDEVIEW VS200 research slide scanner (OLYMPUS). The neuropil density of the suprapyramidal region at the level of CA1, CA3 associational connections and DG within the hippocampus were quantitatively analyzed. Similarly, regions of the association cortex, primary somatosensory cortex and piriform cortex were analyzed for cortical neuropil density.

#### Behavior

Assessment of animal behavior commenced 8 weeks post-infection (approximately 12 weeks of age for neonatally infected mice and 18 weeks of age for adult infected mice). All behaviors were assessed between 10:00 and 15:00 of the light cycle in a designated light-controlled room within a category-II containment facility. All equipment and surfaces were cleaned carefully with 70% ethanol between mice to avoid olfactory cues. The order of behavioral assessments was as follows: repetitive behavior (e.g., marble burying, open-field activity, grooming and rearing) followed by social interaction and finally, tests of learning and memory (e.g., novel object recognition).

#### Marble burying

Mice were assessed for repetitive digging behaviors using the marble burying task. Mice were placed in a rectangular cage (48 cm long, 37 cm wide) with 5 cm of bedding leveled at the base. 30 glass marbles were laid on top of the bedding, interspersed uniformly in a 5 x 6 grid arrangement. The latency to commence digging and the number of marbles buried (>50% covered by bedding) was assessed over 30 min.

#### Open-field activity

General locomotor activity in a novel open-field environment was assessed using ANY-maze video-tracking system. For all open field locomotor activity experiments, mice were moved from the housing room and left in the test room for 15 min prior to beginning the task to ensure an optimal state of arousal. Mice were individually placed in the open-field arena (60 cm long, 40 cm wide with four walls of 19 cm height) and a top-mounted camera was placed approximately 1 m above the cages to record 5 min testing sessions. The floor of the box was divided into a grid of equal-sized squares. The number of squares crossed over a 5 min test session was measured.

#### Repetitive self-grooming and rearing

Spontaneous repetitive self-grooming and rearing behavior were assessed. Each mouse was habituated to the behavioral arena for 5 min and was then scored for cumulative time spent grooming all body regions during the second 5 min of the test session. The number of rears performed during this test session was also quantified during the second 5 min of the test session. Subject animals standing on both hind paws in a vertical upright position was considered rearing. Scoring was performed from coded videos by a trained investigator blind to experimental treatment groups.

#### Social interaction

The duration of social interaction was assessed. Subject mice were placed in a plexiglass cage opposite to a novel, age- and sex-matched mouse separated by a perforated partition. The duration of reciprocal time spent interacting with the novel, age- and sex-matched mouse at the perforated partition was recorded over a 5 min test session.

#### Novel object recognition

The novel object recognition (NOR) test was conducted to assess learning and memory and consisted of a habituation day, training day and testing day as previously described.^69^ Mice were initially exposed to the experimental apparatus in the absence of objects for 5 min (habituation). 24 h after habituation, mice were then exposed to 2 identical objects placed 5 cm away from the walls of the arena for 5 min (training). After a 24 h retention interval, one of the previously explored familiar objects was replaced with a novel object and the amount of time mice spent exploring the novel object versus the familiar object was measured (test).

### Quantification and Statistical analysis

Statistical analysis was performed using Prism 9 (GraphPad Software). Data were analyzed using the Student’s unpaired 2-tailed t-test for independent means. Data are expressed as the mean ± SEM. Values of ∗*p* < 0.05 were considered significant. All dots on plots represent biological replicates or individual cells. Details provided in figure legends.
