# Supplementary material for: Neonatal infection with Bordetella pertussis promotes autism-like phenotypes in mice
Source: iScience. 2024 Dec 9;28(1):111548. doi: 10.1016/j.isci.2024.111548 (PMC11784780; doi:10.1016/j.isci.2024.111548)
Supplement: Document S1. Figures S1–S11 [file mmc1.pdf]

iScience, Volume *28*

Supplemental information

Neonatal infection with *Bordetella pertussis* promotes autism-like phenotypes in mice

Eoin O'Neill, Lucy Curham, Caitlín Ní Chasaide, Síofra O'Brien, Gavin McManus, Barry Moran, Keith Rubin, Steven Glazer, Marina A. Lynch, and Kingston H.G. Mills

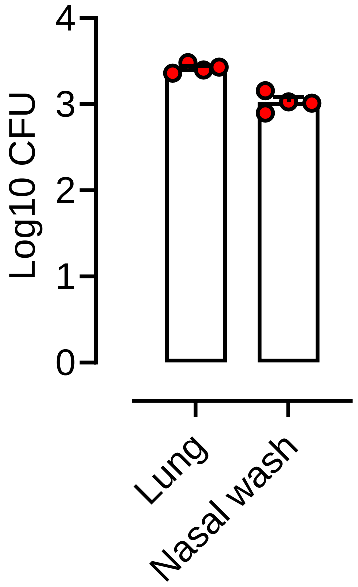

Figure S1. CFU counts in respiratory tract following aerosol challenge of neonatal mice with *B. pertussis*.

Ten-day old neonatal mice were aerosol challenged with live *B. pertussis*. CFU counts were enumerated in the lung and nasal wash 4 hours post-aerosol challenge with *B. pertussis.* Data are presented as mean ± SEM. *n* = 4 from one experiment.

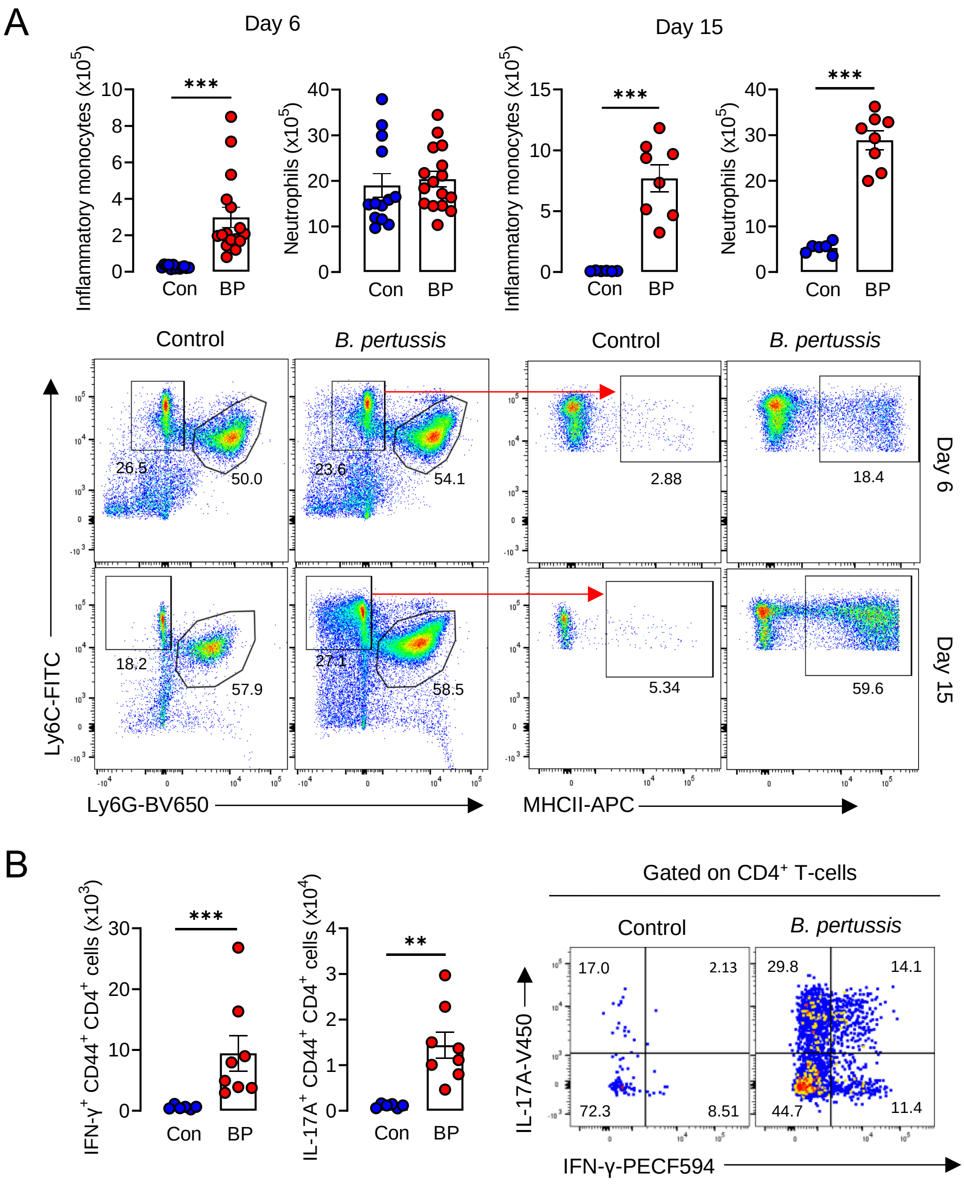

Figure S2. Nasal inflammation following respiratory challenge of neonatal mice with *B. pertussis*.

Ten-day old neonatal mice were aerosol challenged with live *B. pertussis*.

(A) Number of inflammatory monocytes ($MHCII^+Ly6C^+CD11b^+$) and neutrophils ($Ly6c^+LY6G^+$) in the nose 6 and 15 days post-challenge with sample flow cytometry plots below.

(B) Number of IFN-γ- or IL-17A-secreting $CD4^+$ T cells in the nose by intracellular cytokine staining and flow cytometry, with sample flow plots to the right. Data are presented as mean ± SEM n = 13-16 for Day 6 flow cytometry (controls: 5 male and 8 female, *B. pertussis*-infected: 8 male and 8 female) from two independent experiments. n = 6-8 for Day 15 FACS (controls: 4 male and 2 female, *B. pertussis*-infected: 5 male and 3 female). (**P<0.01, ***P<0.001) by Student's unpaired Two-tailed *t*-test.

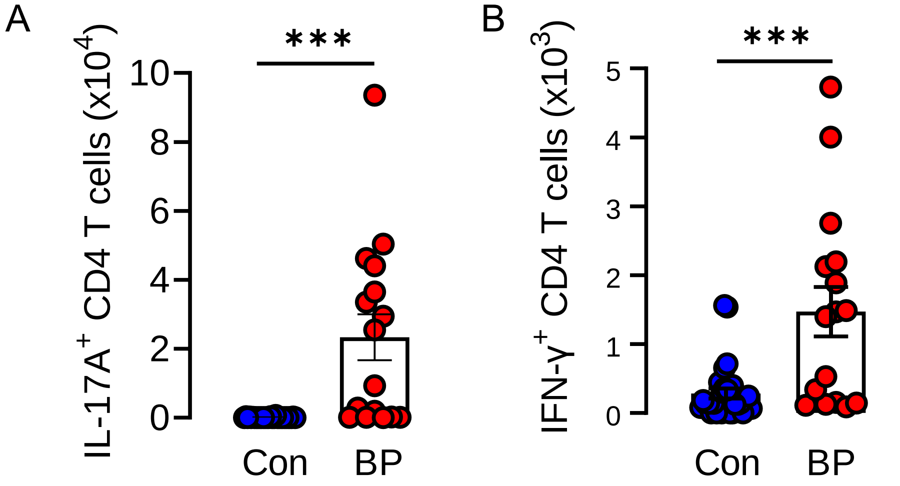

Figure S3. *B. pertussis*-specific IL-17A and IFN-γ-secreting CD4 T cells in the lung following respiratory challenge of neonatal mice with *B. pertussis*.

Ten-day old neonatal mice were aerosol challenged with live *B. pertussis*. Ten weeks later, mice were euthanized and *B. pertussis*-specific Th1 and Th17 responses in the lung were analysed by intracellular cytokine staining and flow cytometry. Number of *B. pertussis*-specific IL-17A-secreting (A) or IFN-γ-secreting (B) CD4 T cells in the lung by intracellular cytokine staining and flow cytometry. Data are presented as mean ± SEM n = 16-30 from two independent experiments (controls: 12 male and 18 female, *B. pertussis*-infected: 8 male and 8 female). (***P<0.001) by Student's unpaired Two-tailed *t*-test.

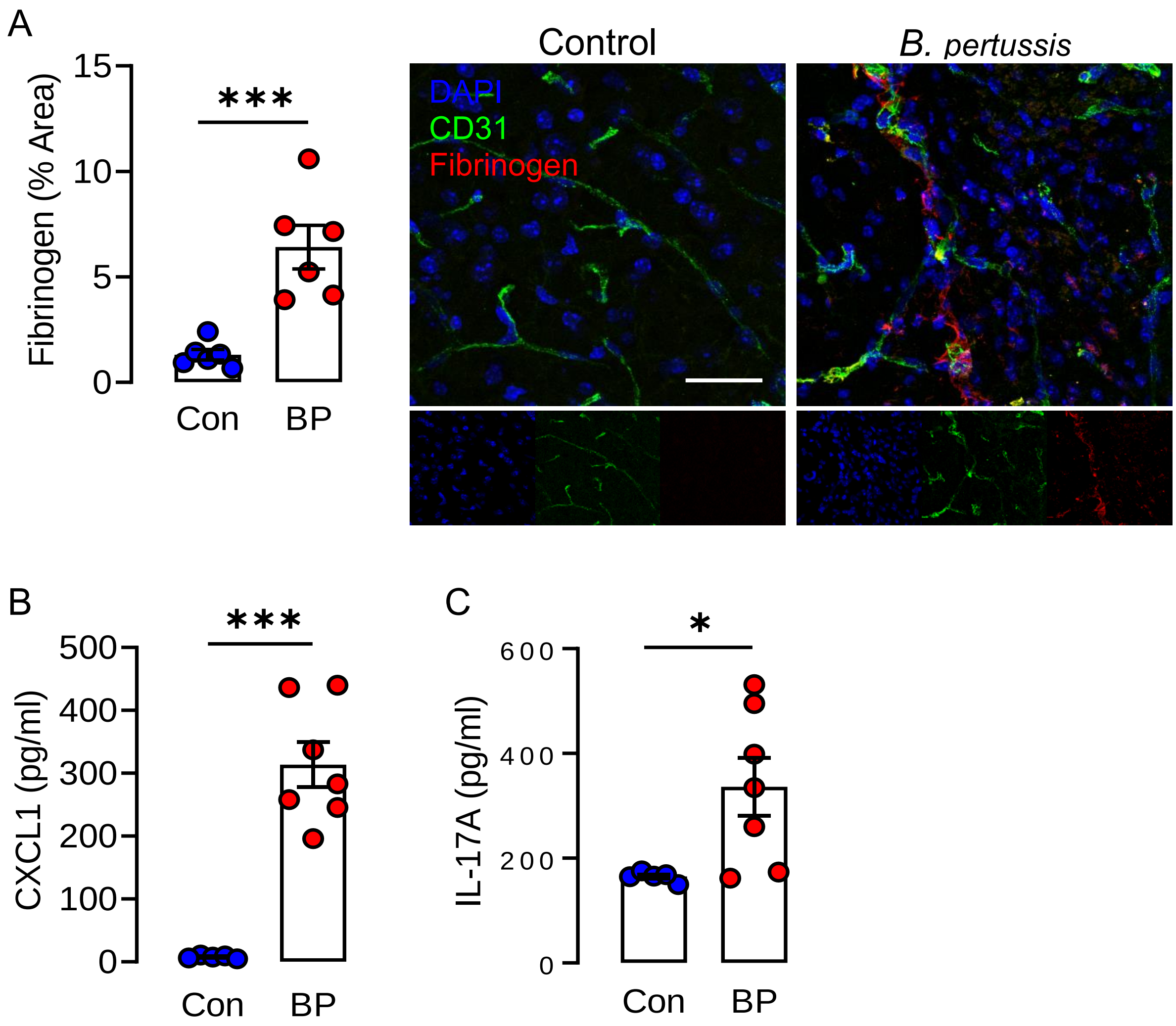

Figure S4. Increased blood-brain barrier permeability and CXCLI and IL-17A concentrations in the brain 15 days post-aerosol challenge with *B. pertussis*.

Ten-day old neonatal mice were aerosol challenged with live *B. pertussis*.

(A) Fibrinogen deposition in the brain 15 days after neonatal infection, with representative confocal images. Scale bar, 100 µm.

(B) CXCL1 concentrations in brain 15 days post challenge with *B. pertussis*.

(C) IL-17A concentrations in brain 15 days post challenge with *B. pertussis*.

Data are presented as mean ± SEM n = 5-7 (controls: 3 male and 2 female, *B. pertussis*-infected: 4 male and 3 female). (*p < 0.05, ***P<0.001) by Student's unpaired Two-tailed *t*-test.

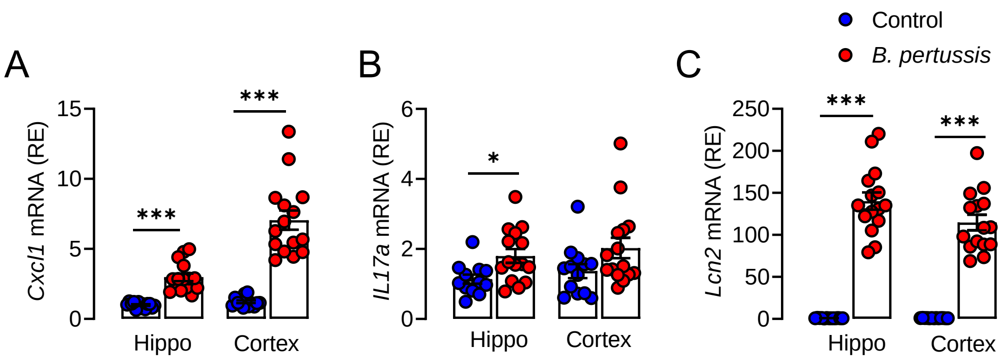

Figure S5. Enhanced *Cxcl1 IL17a* and *Lcn2* expression in the brain following infection of neonatal mice with *B. pertussis*.

Neonatal mice (P10) were aerosol infected with live *B. pertussis.*

(A) *Cxcl1* mRNA expression in hippocampus and cortex 6 days after neonatal challenge with *B. pertussis.*

(B) *IL17a* mRNA expression in hippocampus and cortex 6 days after neonatal challenge with *B. pertussis.*

(C) *Lipocalin-2 (Lcn2)* mRNA expression in hippocampus and cortex 6 days after neonatal challenge with *B. pertussis*.

Data are presented as mean ± SEM *n* = 13-15 (controls: 6 male and 7 female, *B. pertussis*-infected: 9 male and 6 female). (*P<0.05, ***P<0.001) by Student's unpaired Two-tailed t-test.

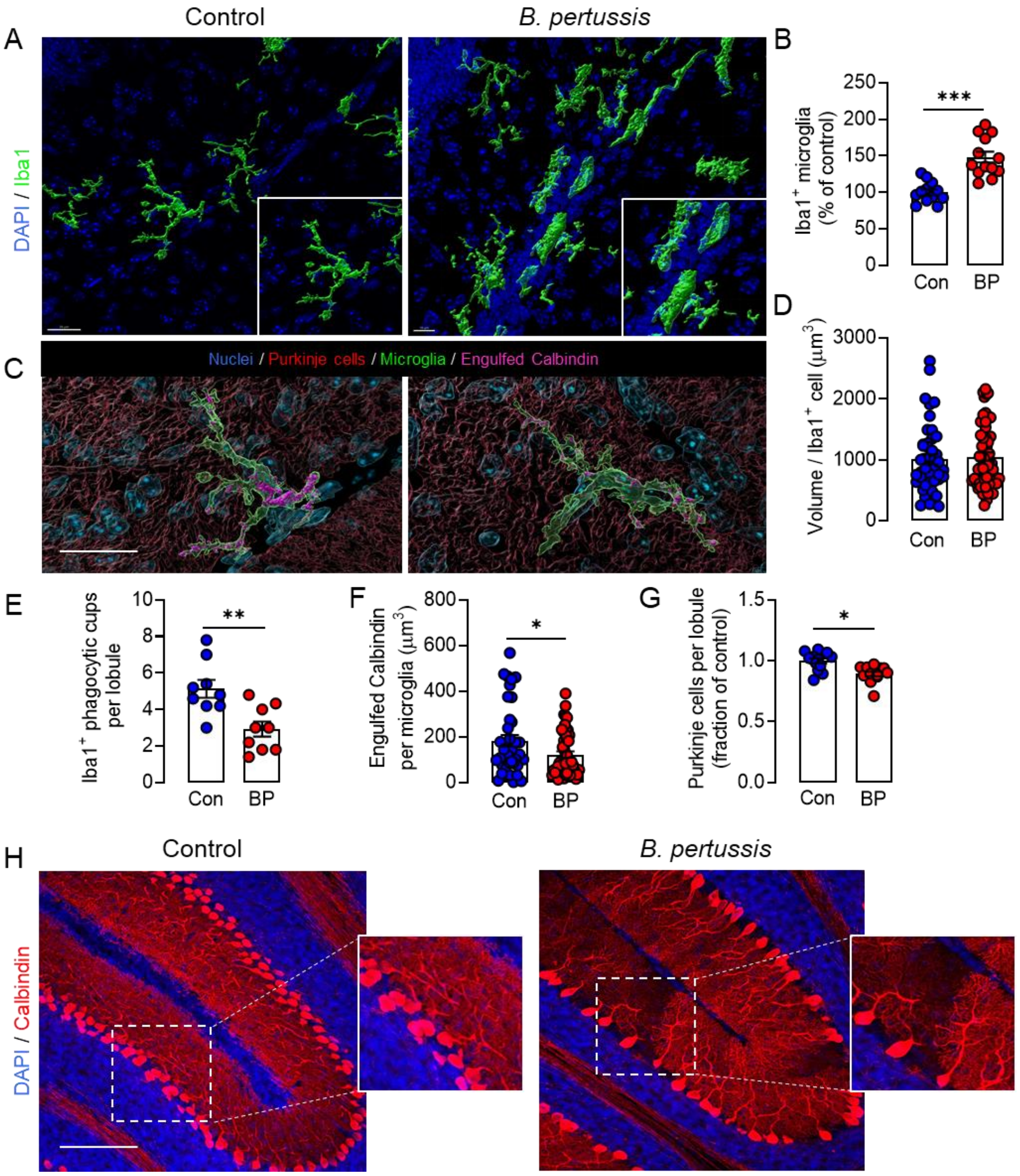

Figure S6. Neonatal infection with *B. pertussis* impairs Purkinje cell dendrite remodelling in the developing cerebellum.

Ten-day old neonatal mice were aerosol challenged with live *B. pertussis*.

(A) Representative Iba1 staining and Imaris reconstructions of microglia in cerebellum. Scale bar, 50 µm.

(B) Number of $Iba1^{+}$ cells in cerebellum 6 days after neonatal aerosol challenge with *B. pertussis*.

(C) Representative Imaris reconstructions of $Iba1^{+}$ microglia (green), $calbindin^{+}$ Purkinje cells (red), engulfed calbindin (pink) and $DAPI^{+}$ nuclei (blue). Scale bar, 10 µm.

(D) Volume of individual Iba1 cells in cerebellum.

(E) Quantification of $Iba1^{+}$ phagocytic cups per cerebellar lobule.

(F) Volume of engulfed calbindin inside $Iba1^{+}$ microglia.

(G) Quantification of $calbindin^{+}$ Purkinje cells per cerebellar lobule. (H) Representative images of $calbindin^{+}$ Purkinje cells in cerebellum. Scale bar, 100 µm.

Data are presented as mean ± SEM for confocal, $n = 9$ (controls: 4 male and 5 female, *B. pertussis*-infected: 4 male and 5 female) from 2 independent experiments & for Imaris reconstructions $n = 50$-63 individual cells. (*$p < 0.05$, **$p < 0.01$, ***$p < 0.001$) by Student's unpaired 2-tailed t-test.

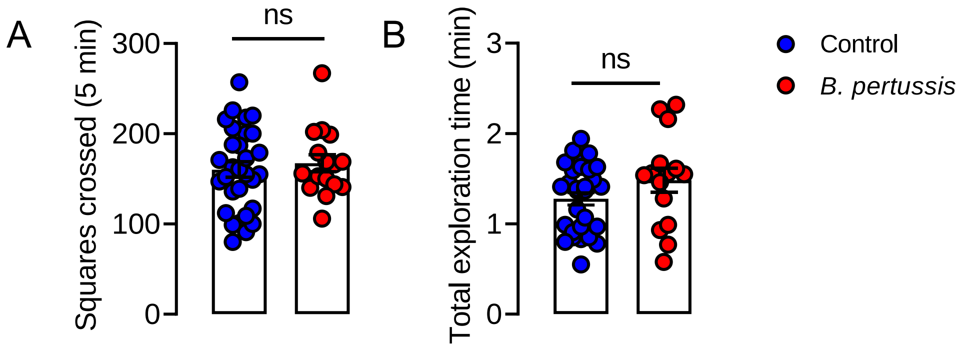

Figure S7. General locomoter and exploratory behaviour in response to neonatal infection with *B. pertussis*.
Neonatal mice (P10) were aerosol infected with live *B. pertussis* and behaviour was assessed 10 weeks later.
(A) Squares crossed in open field.
(B) Total exploration time of novel and familiar object in NOR task.
Data are presented as mean ± SEM. n = 16-30 from two independent experiments (controls: 12 male and 18 female, *B. pertussis*-infected: 8 male and 8 female), by Student's unpaired Two-tailed t-test.

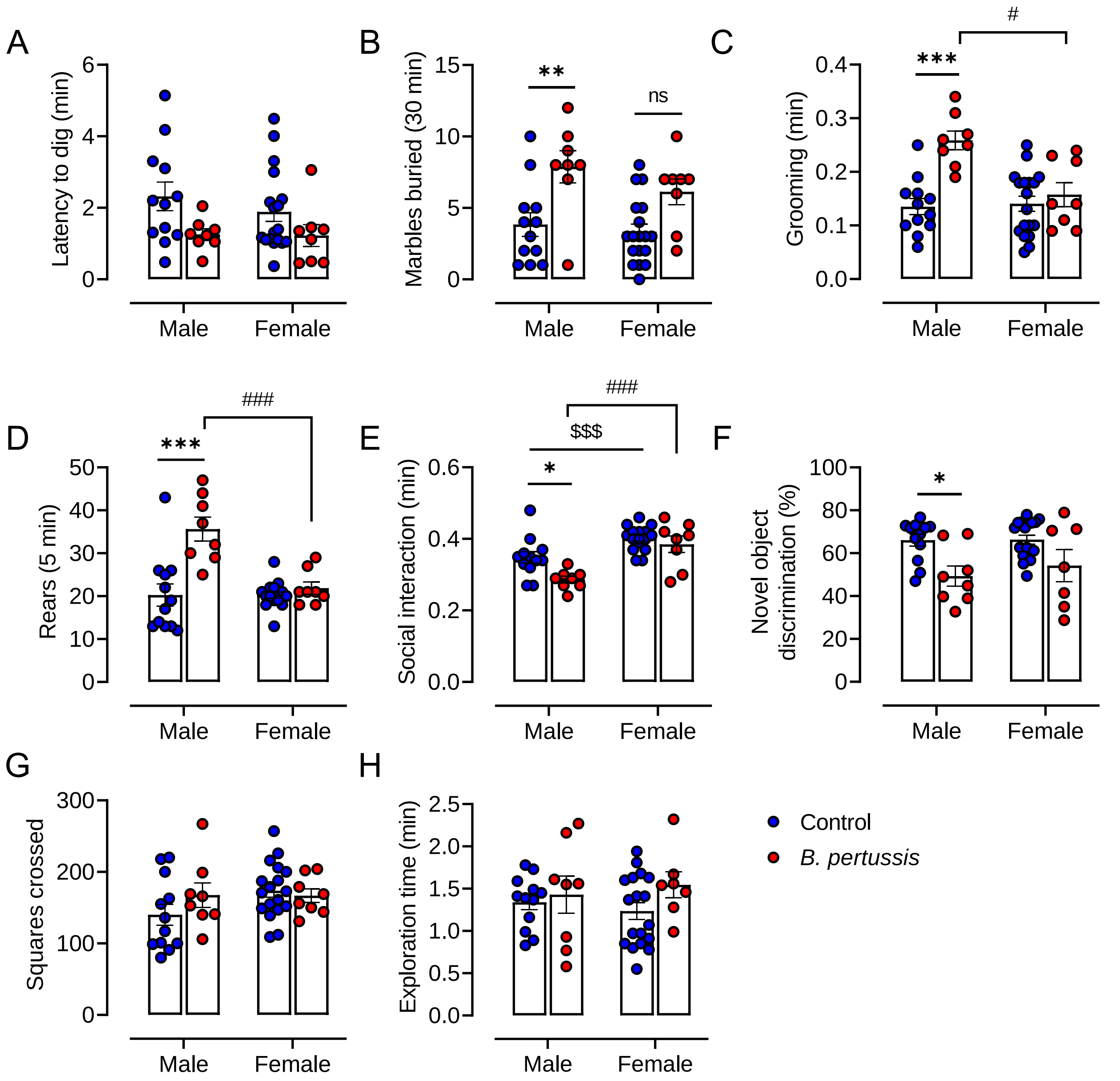

Figure S8. Sex differences in ASD-like behaviour in response to neonatal infection with *B. pertussis.*

Neonatal mice (P10) were aerosol infected with live *B. pertussis* and stereotypic repetitive behaviour, restricted interests and social interaction was assessed ten weeks later in adulthood.

(A) Latency to commence digging in marble burying test.

(B) Number of marbles buried over 30 minutes in the marble burying test.

(C) Duration of grooming over a 5 min test session.

(D) Number of rears performed over a 5 min test session.

(E) Duration of social interaction with a novel, age- and sex-matched mouse over a 5 min test session.

(F) Novel object discrimination index in the novel-object recognition (NOR) task.

(G) Number of squares crossed in the open field.

(H) total exploration time in NOR.

Data are presented as mean ± SEM. n = 8-18 from two independent experiments (Controls: 12 male and 18 female, *B. pertussis*-infected: 8 male and 8 female). (*$p < 0.05$, **$p < 0.01$, ***$p < 0.001$) by Two-way ANOVA followed by Tukey *post hoc* test.

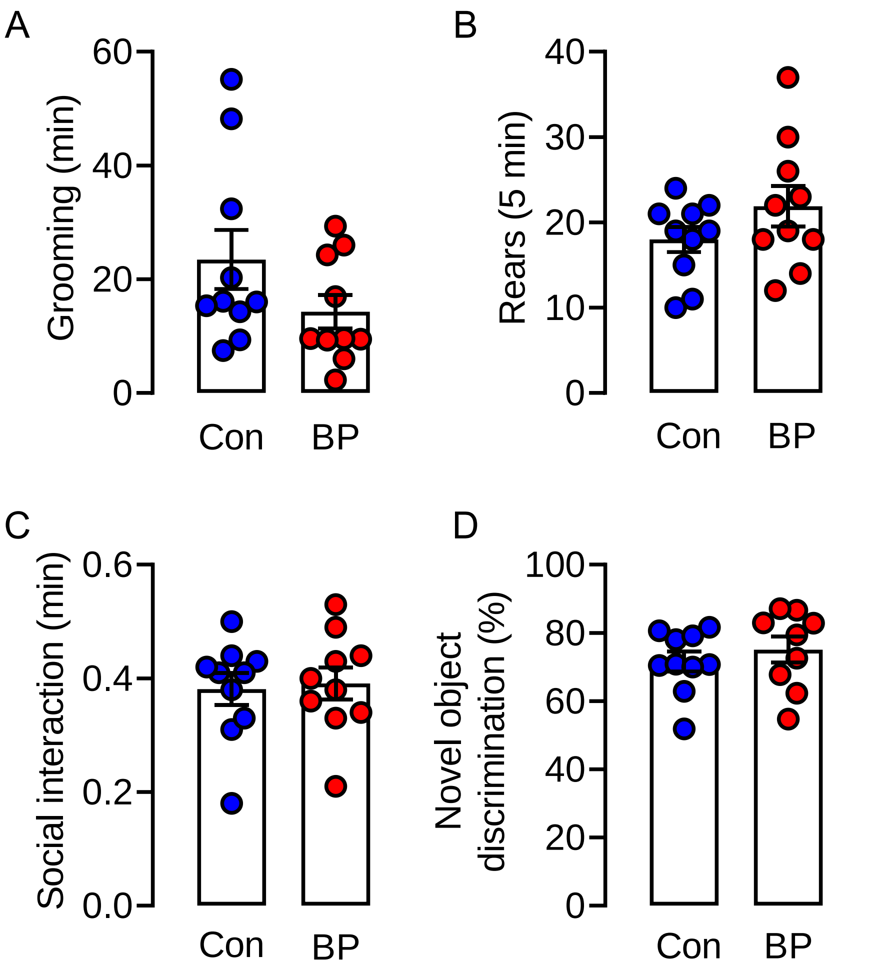

Figure S9. Infection of adult mice with *B. pertussis* does not enhance ASD-like behaviour.

Eight-week old adult mice were aerosol infected with live *B. pertussis* and stereotypic repetitive behaviour, social interaction and restricted interests were assessed 10 weeks later.

(A) Duration of grooming over a 5-minute test session.

(B) Number of rears performed over a 5 min test session.

(C) Duration of social interaction with a novel, age- and sex-matched mouse over a 5 min test session.

(D) Novel object discrimination index in the novel-object recognition task.

Data are presented as mean ± SEM. *n* = 10 (Controls: 5 male and 5 female, *B. pertussis*-infected: 5 male and 5 female). by Student's unpaired Two-tailed t-test.

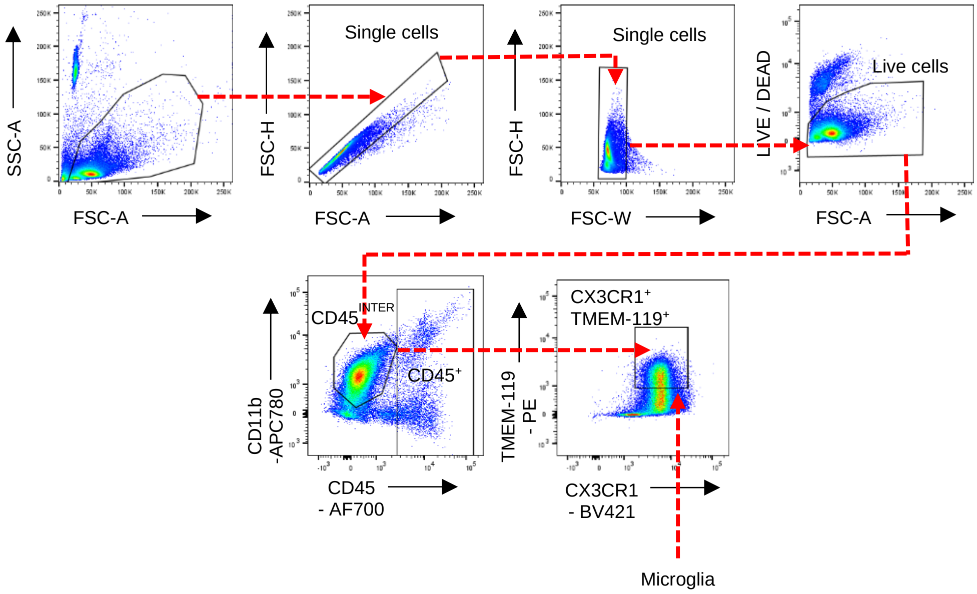

Figure S10. Flow cytometry gating strategy for brain tissue cells.

Gating strategy for identification of microglia ($CD45^{INTER}CD11b^{+}CX3CR1^{+}TMEM\text{-}119^{+}$) in brain.

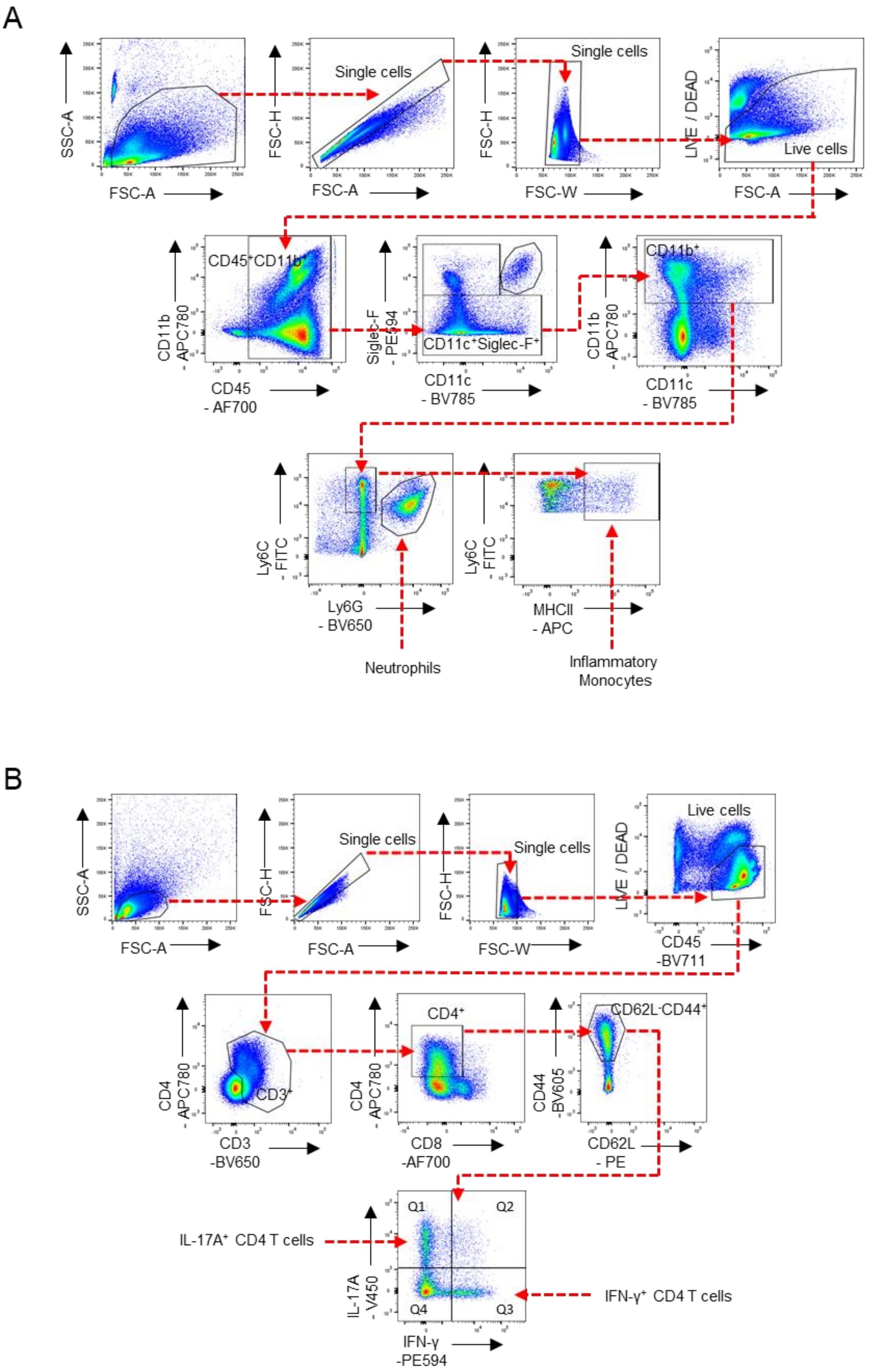

Figure S11. Flow cytometry gating strategy for lung tissue cells.

(A) Gating strategy for identification of neutrophils (CD45$^+$CD11b$^+$Ly6G$^+$) and inflammatory monocytes (CD45$^+$CD11b$^+$LY6C$^+$MHCII$^+$) in lung.

(B) Gating strategy for identification of IL-17A- or IFN-γ-secreting activated CD4 T cells (CD45$^+$CD44$^+$CD62L$^-$) in lung.
